# Supplementary figures and images for: GPR168 functions as a tumor suppressor in mouse melanoma by restraining Akt signaling pathway
Source: PLoS One. 2024 May 28;19(5):e0302061. doi: 10.1371/journal.pone.0302061 (PMC11132440; doi:10.1371/journal.pone.0302061)

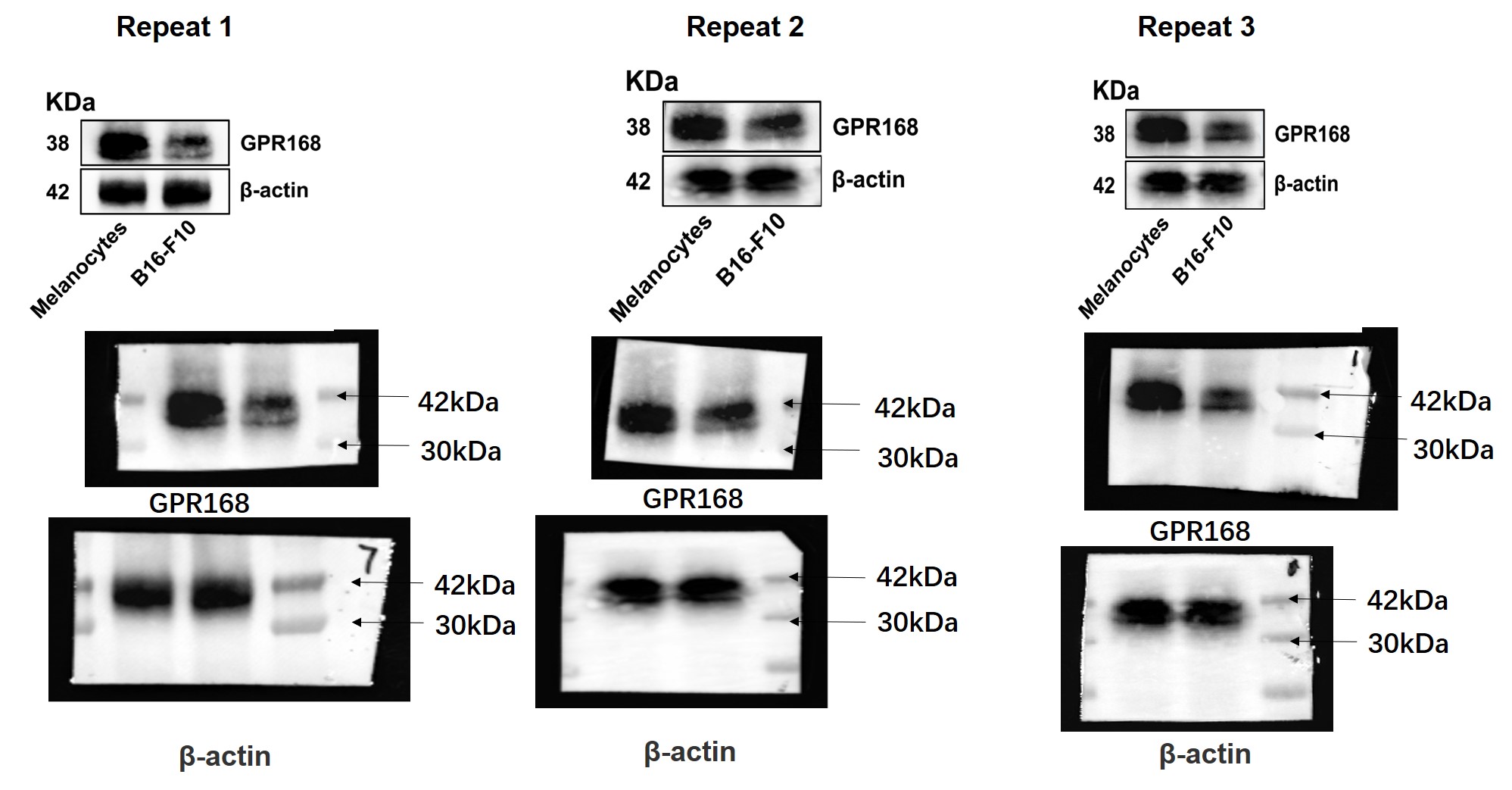

Supplement: S1 Fig — Western blotting results showed the protein expression of GPR168 in mice melanoma cell line B16-F10, compared to normal melanocytes cells (normalized to ß-actin). (JPG) [file pone.0302061.s001.jpg]

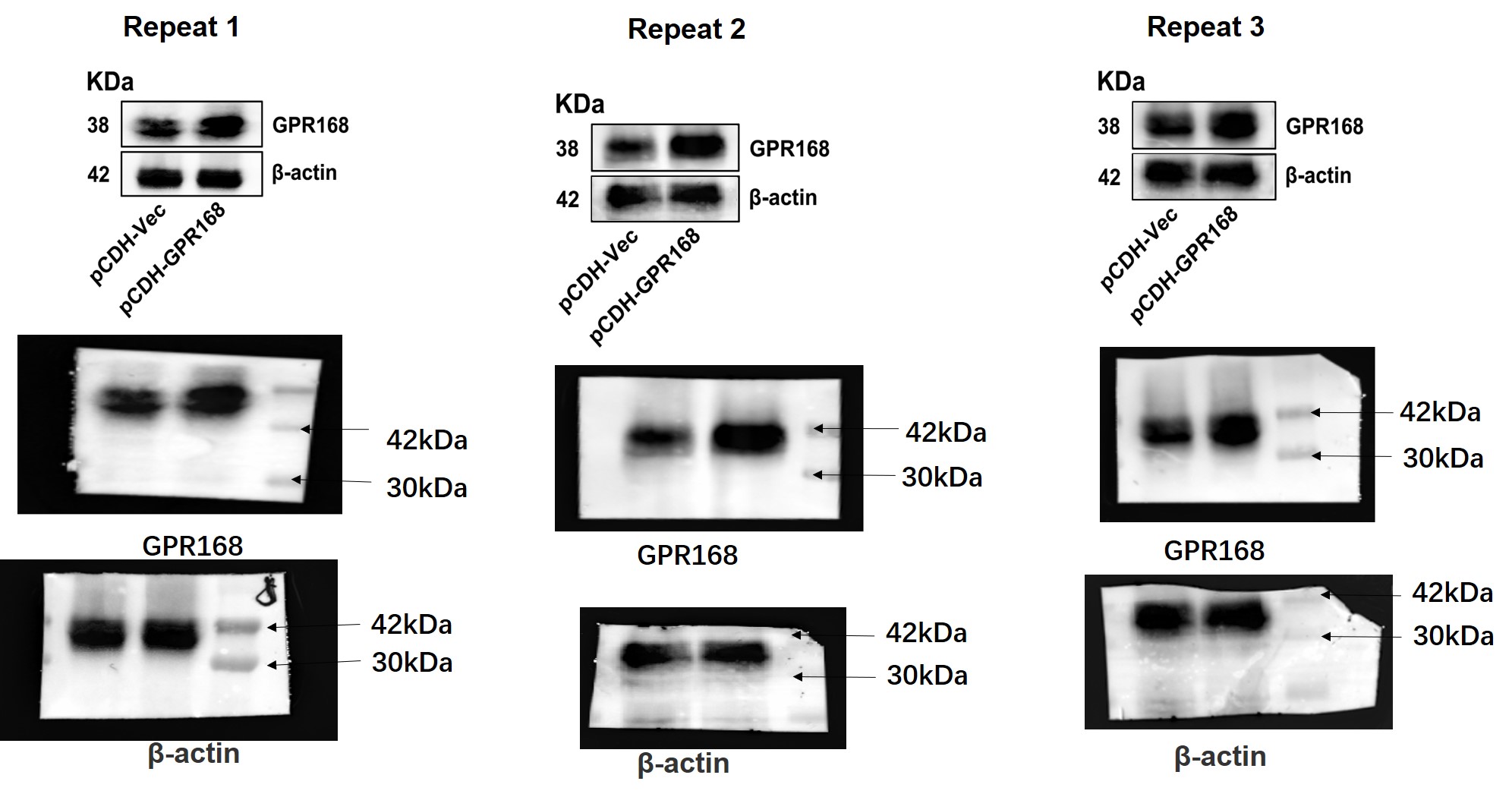

Supplement: S2 Fig — The efficiency of pCDH-GPR168 (GPR168 overexpression) was verified by western blotting, compared to scramble pCDH-Vec. (JPG) [file pone.0302061.s002.jpg]

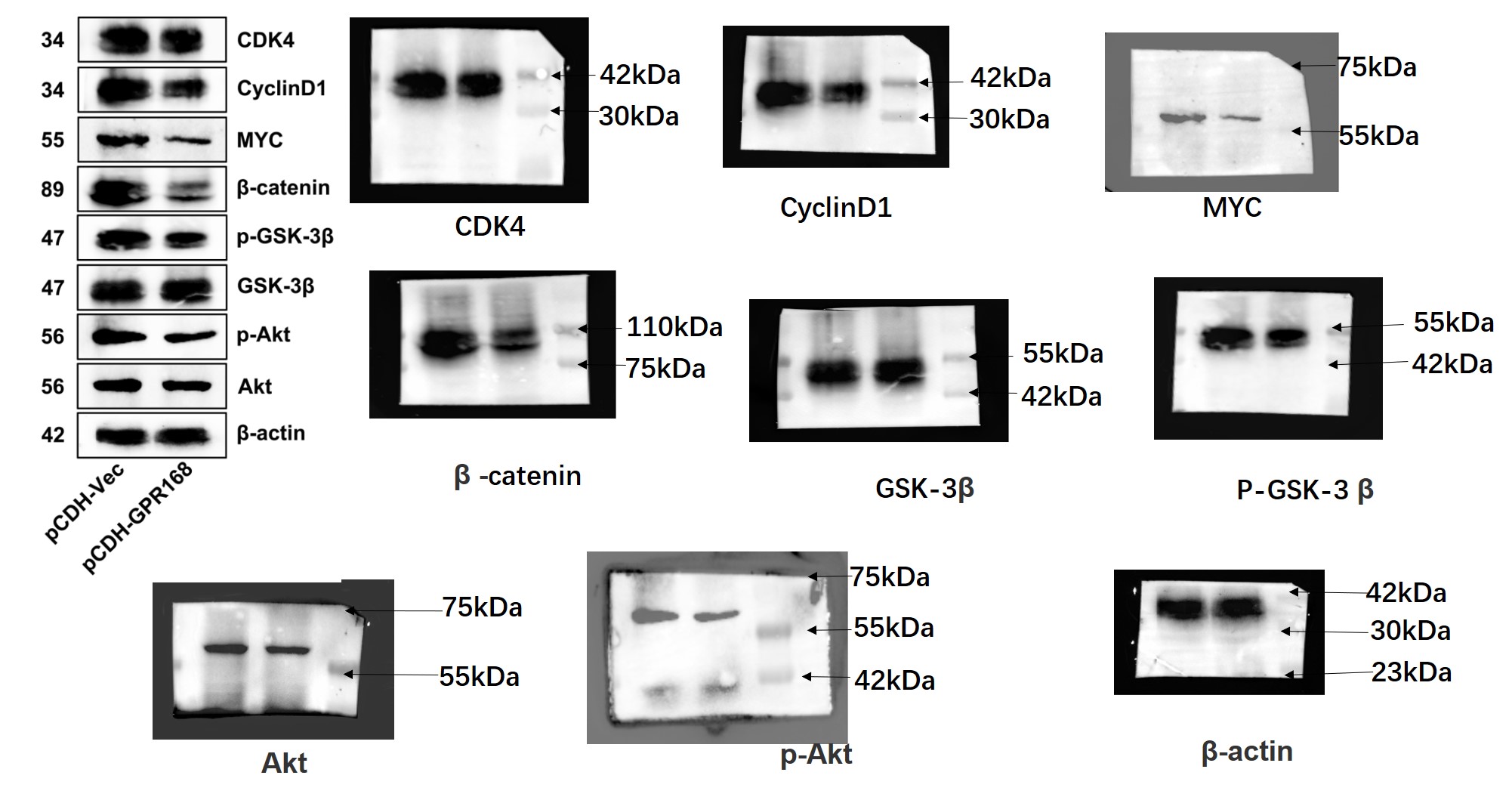

Supplement: S3 Fig — Akt, p-Akt, GSK-3β, p- GSK-3β, β-catenin, Myc, CyclinD1, CDK4 protein expression in pCDH-GPR168 group and pCDH-Vec group. (JPG) [file pone.0302061.s003.jpg]

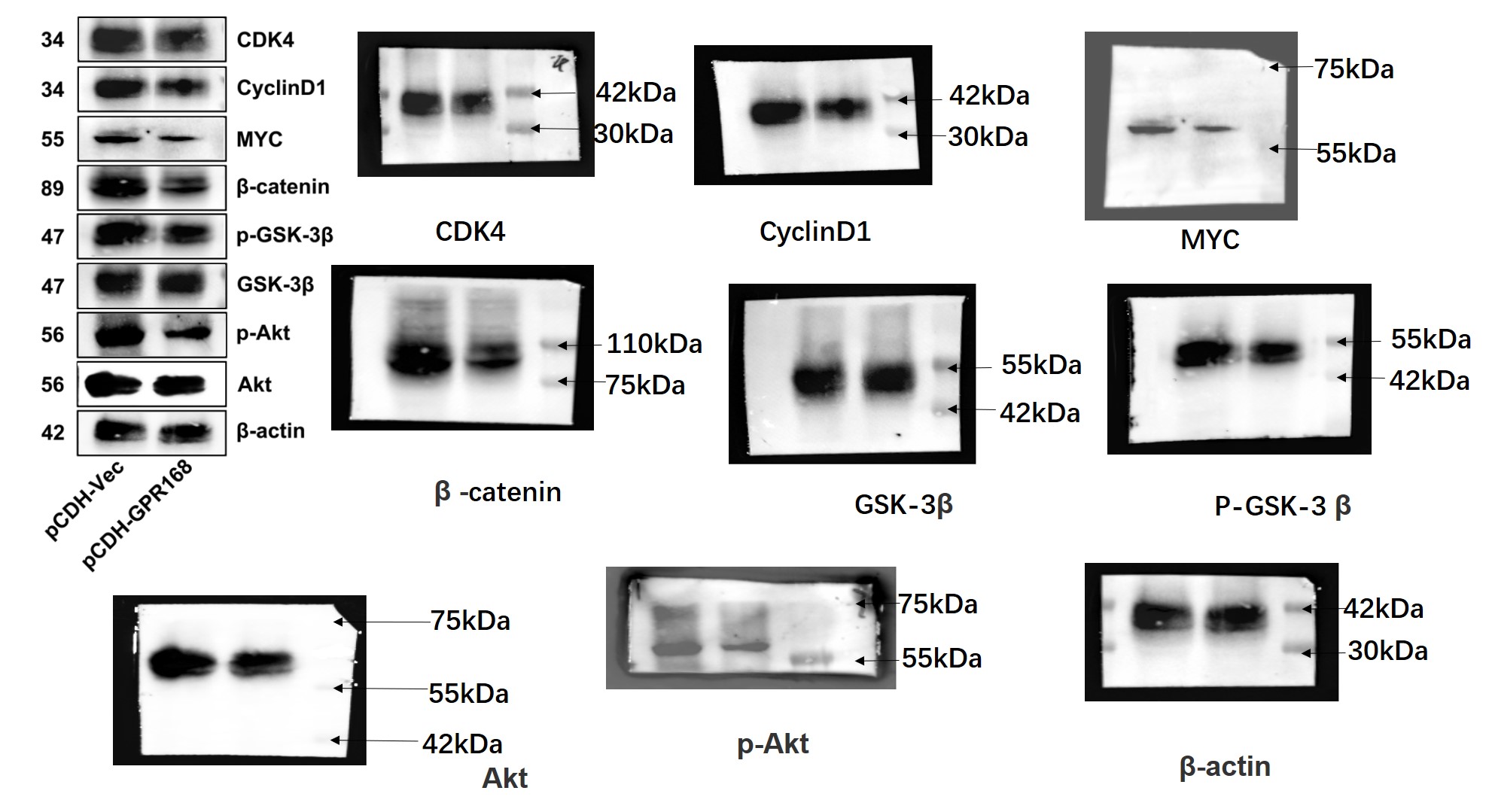

Supplement: S4 Fig — Akt, p-Akt, GSK-3β, p- GSK-3β, β-catenin, Myc, CyclinD1, CDK4 protein expression in pCDH-GPR168 group and pCDH-Vec group. (JPG) [file pone.0302061.s004.jpg]

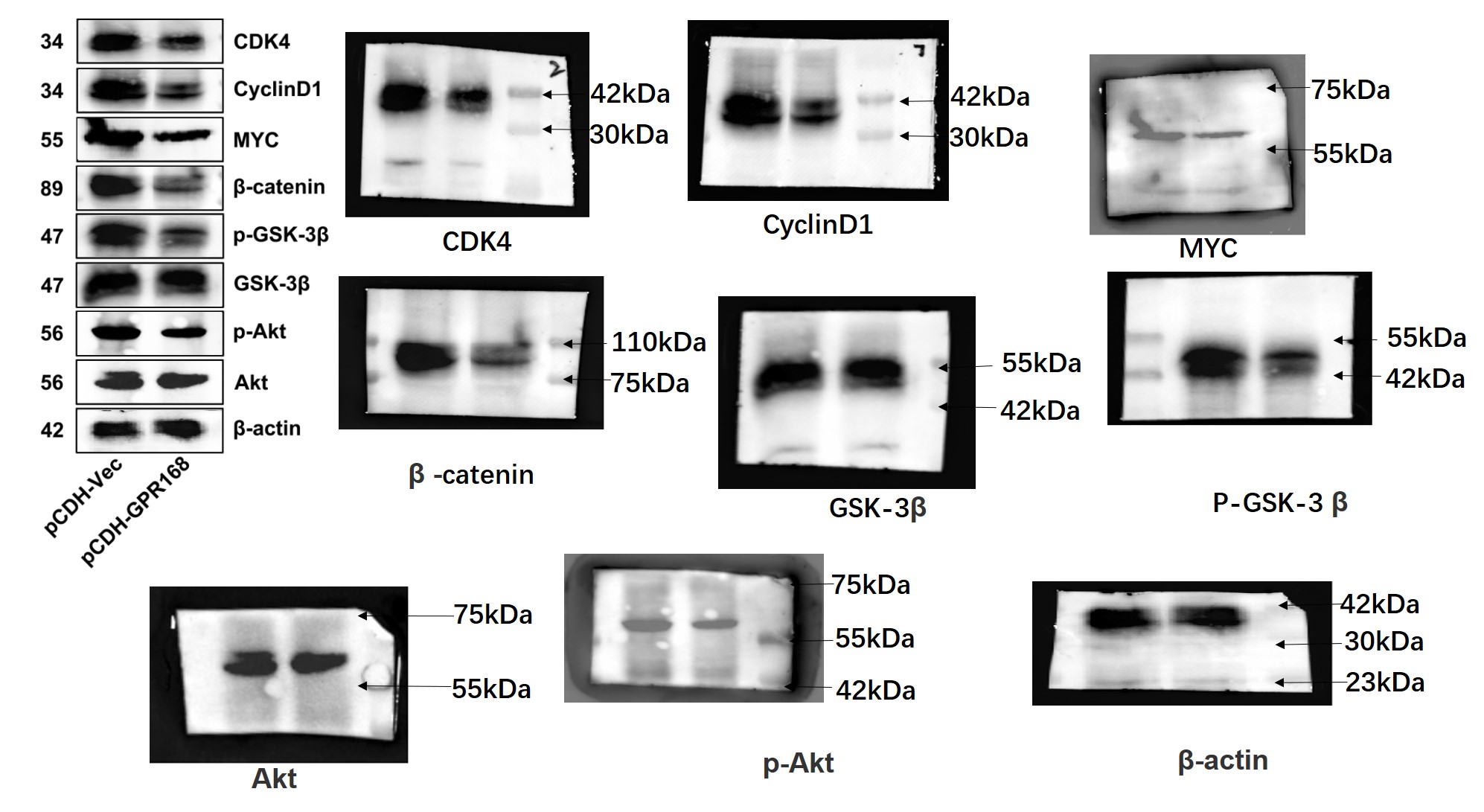

Supplement: S5 Fig — Akt, p-Akt, GSK-3β, p- GSK-3β, β-catenin, Myc, CyclinD1, CDK4 protein expression in pCDH-GPR168 group and pCDH-Vec group. (JPG) [file pone.0302061.s005.jpg]

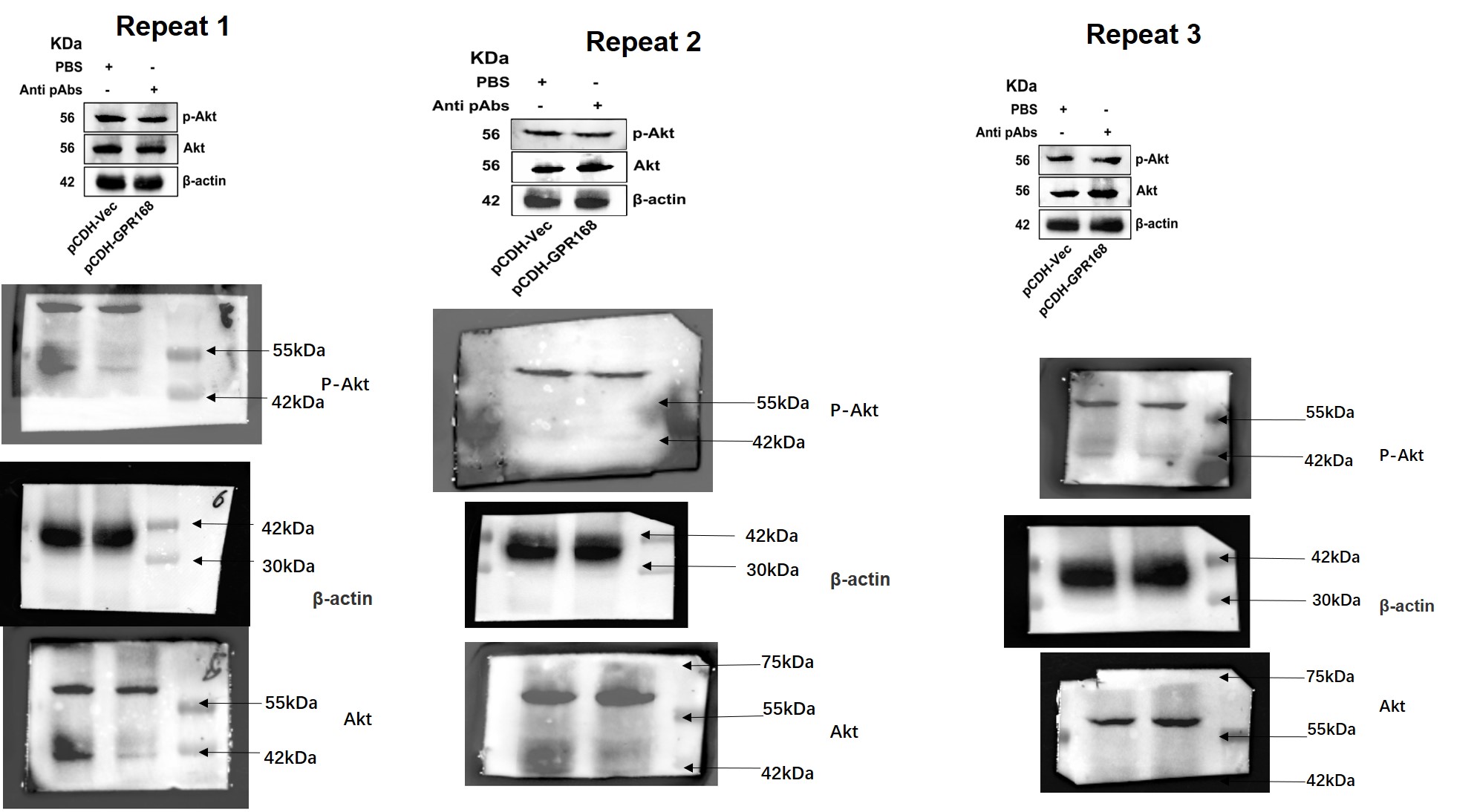

Supplement: S6 Fig — Western botting analysis of Akt and p-Akt protein expression in GPR168 overexpression B16-F10 melanoma cells cultured in 100 ng/ml Anti-GPR168 pAbs medium, pCDH-Vec was set as control. β-actin as normalized protein. (JPG) [file pone.0302061.s006.jpg]
