# Supplementary material for: GPR168 functions as a tumor suppressor in mouse melanoma by restraining Akt signaling pathway
Source: PLoS One. 2024 May 28;19(5):e0302061. doi: 10.1371/journal.pone.0302061 (PMC11132440; doi:10.1371/journal.pone.0302061)
Supplement: S2 Data — (DOCX) [file pone.0302061.s008.docx]

**Minimal data set**

**1. Repetitions and statistics of Immunofluorescence assay in Figure 2D.**


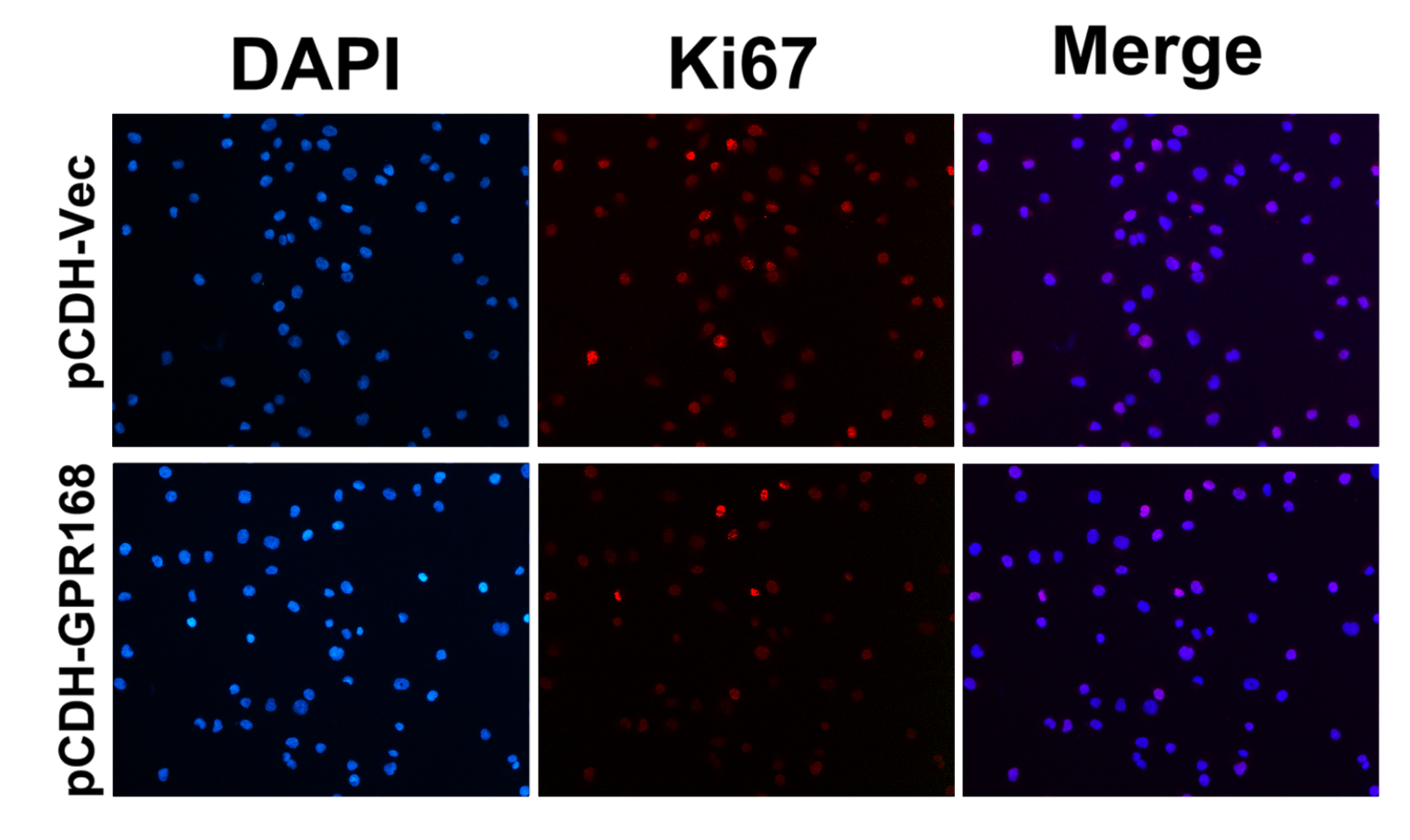

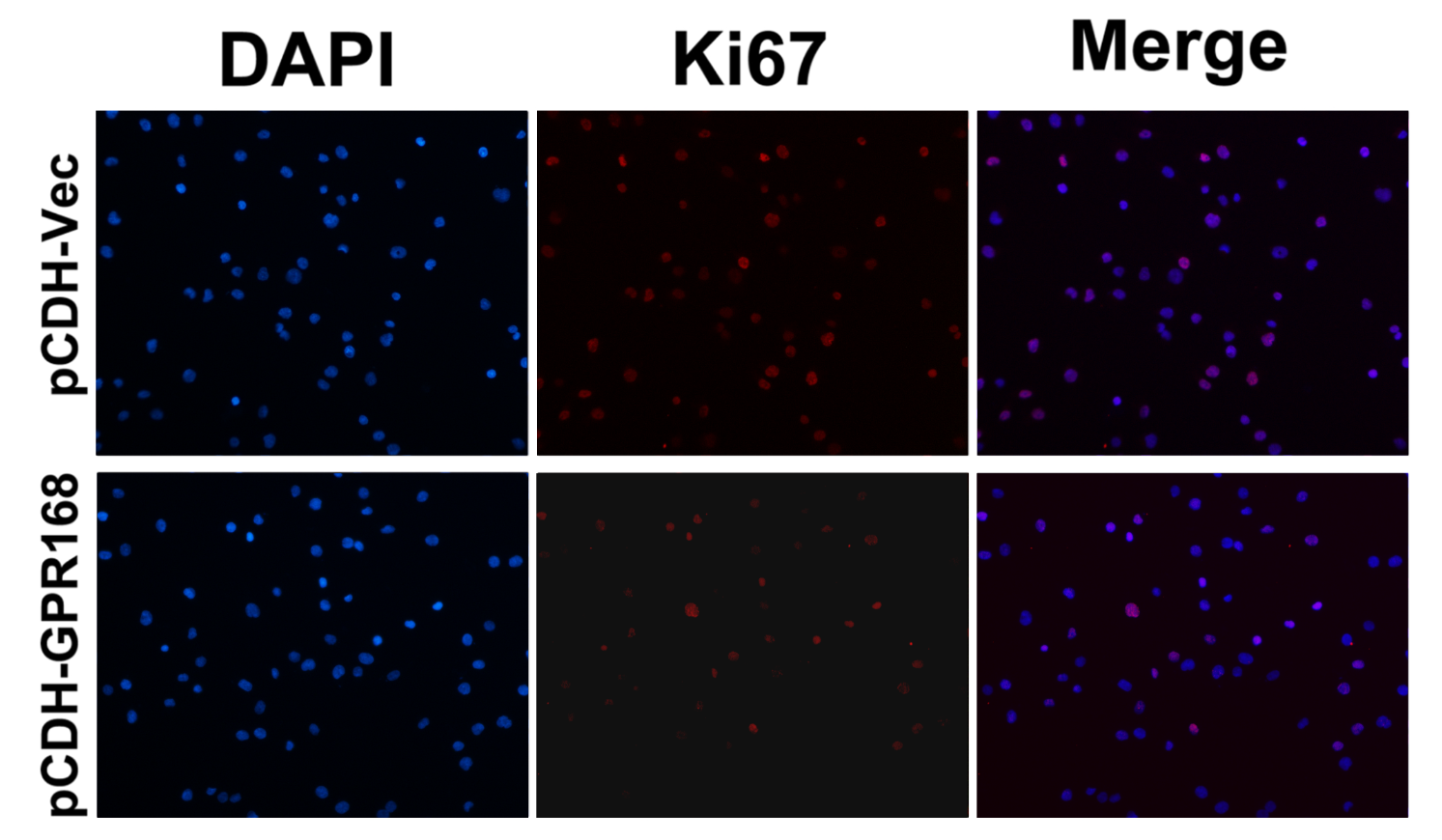

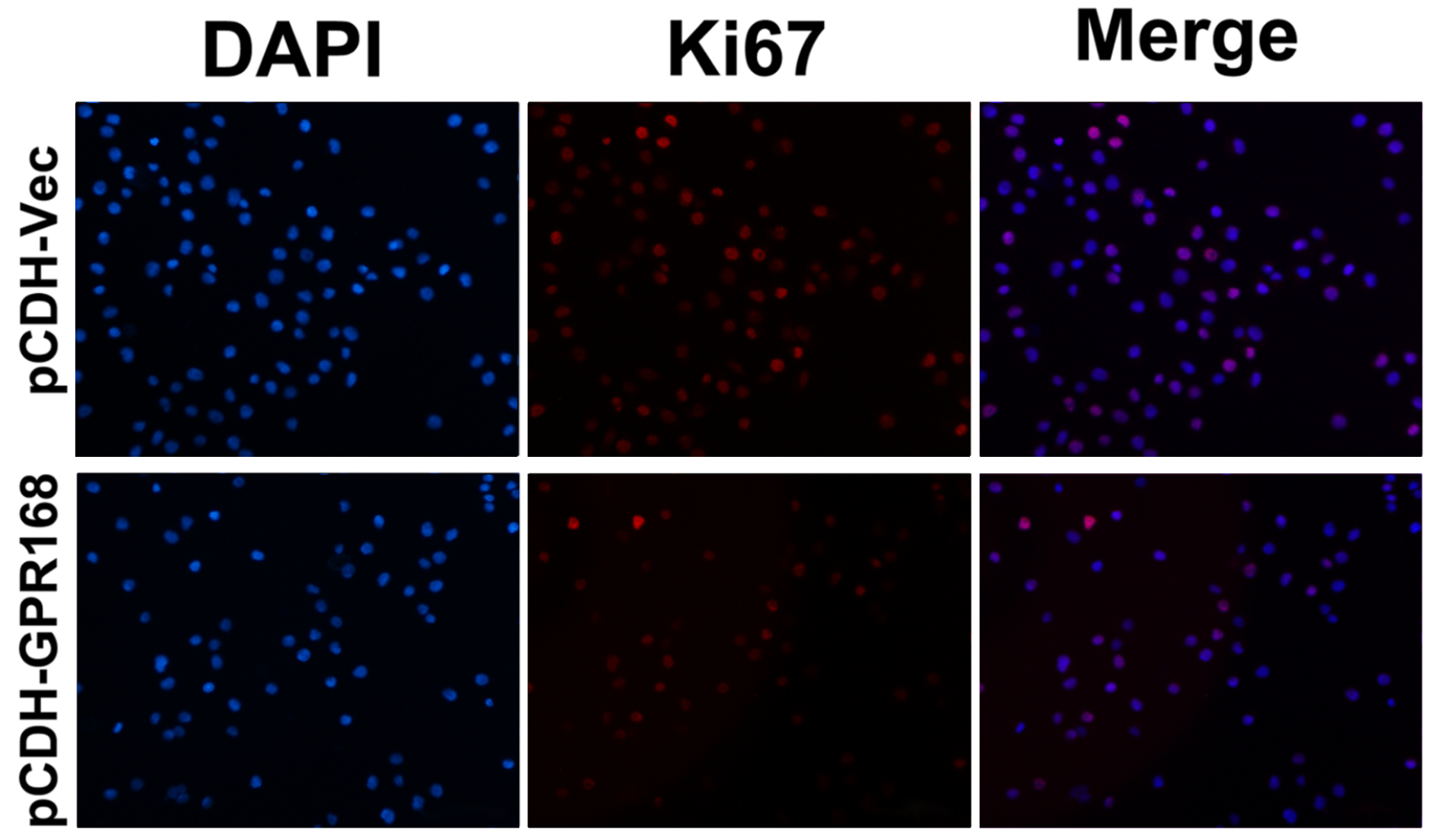

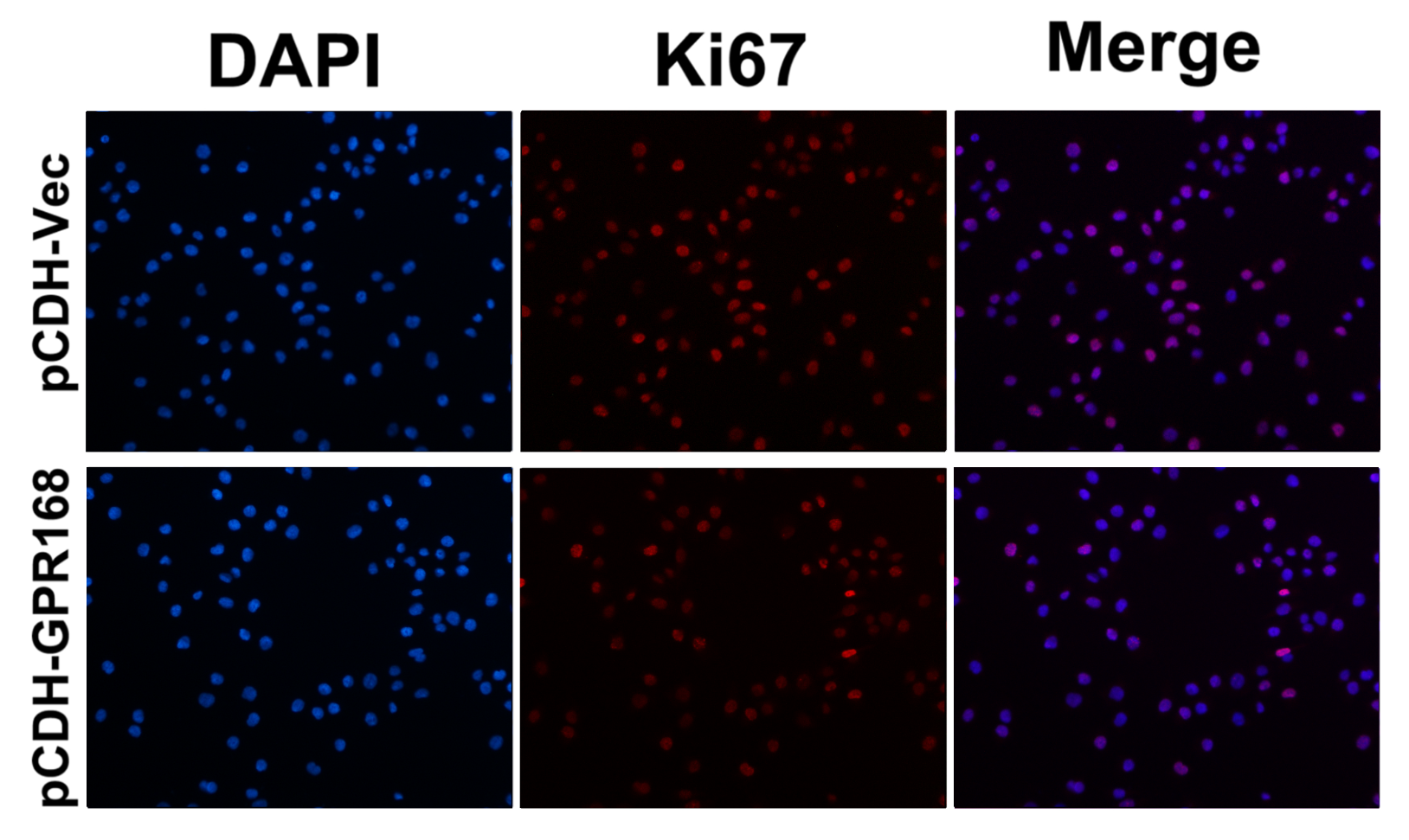


| Statistics (Fig 2D) | Cell | DAPI | Ki67 | 100% |
| --- | --- | --- | --- | --- |
| 1 | pCDH-Vec | 146 | 56 | 38.36 |
|  | pCDH-GPR168 | 151 | 38 | 25.00 |
| 2 | pCDH-Vec | 78 | 32 | 41.03 |
|  | pCDH-GPR168 | 76 | 16 | 21.05 |
| 3 | pCDH-Vec | 67 | 23 | 34.33 |
|  | pCDH-GPR168 | 76 | 21 | 27.63 |
| 4 | pCDH-Vec | 107 | 44 | 41.12 |
|  | pCDH-GPR168 | 76 | 23 | 30.26 |
| 5 | pCDH-Vec | 104 | 37 | 35.57 |
|  | pCDH-GPR168 | 92 | 27 | 29.35 |

**2. Repetitions and statistics of transwell assay in Figure 2H.**


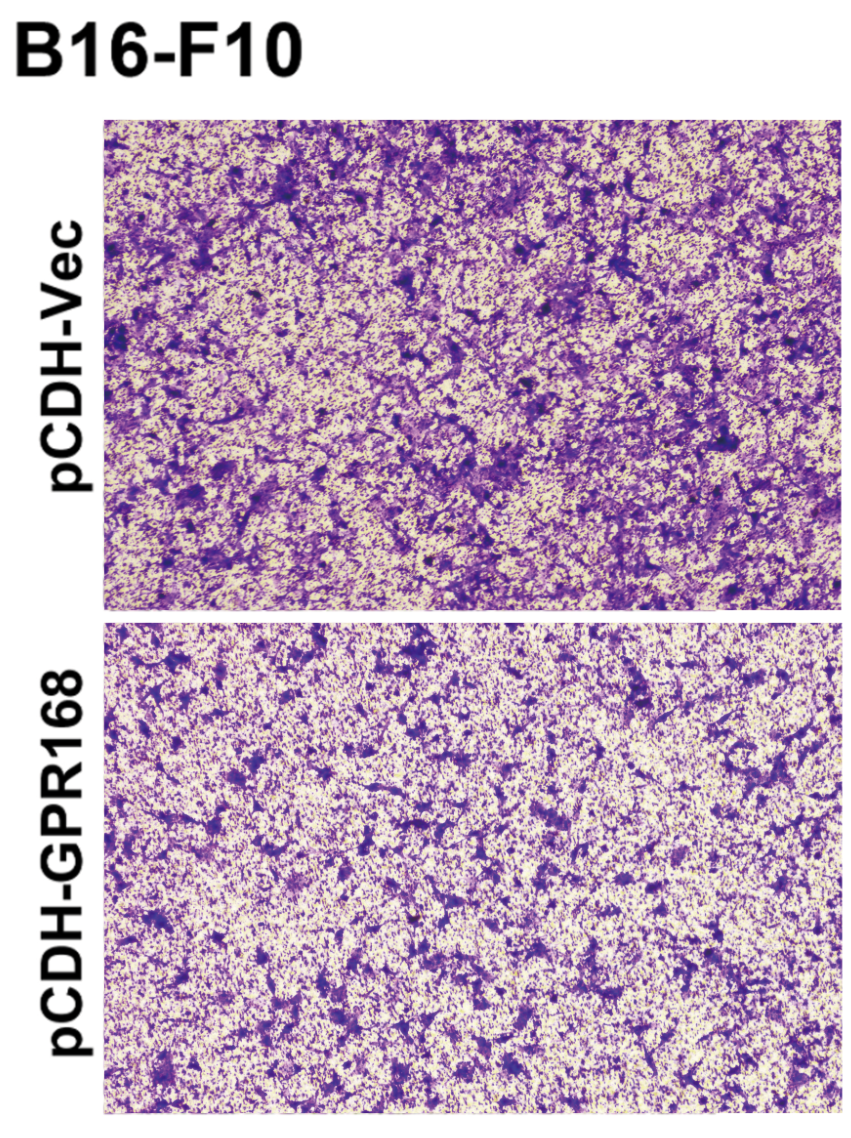

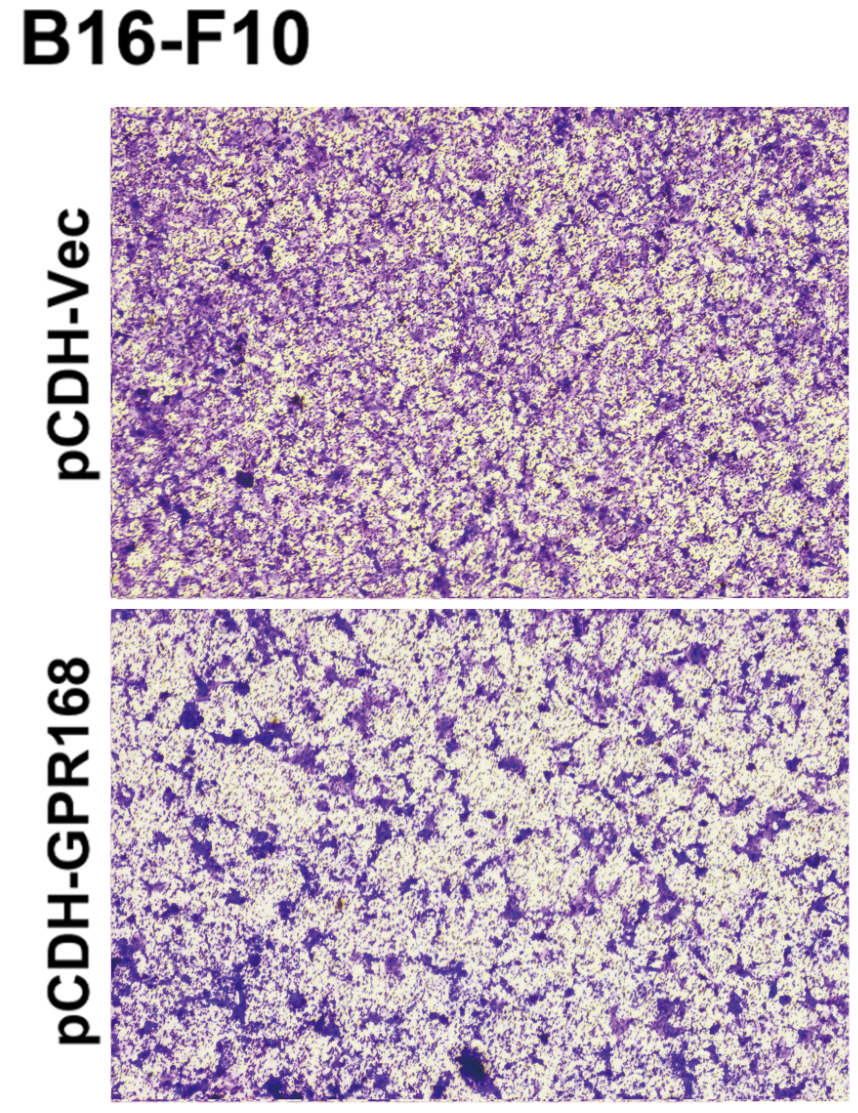

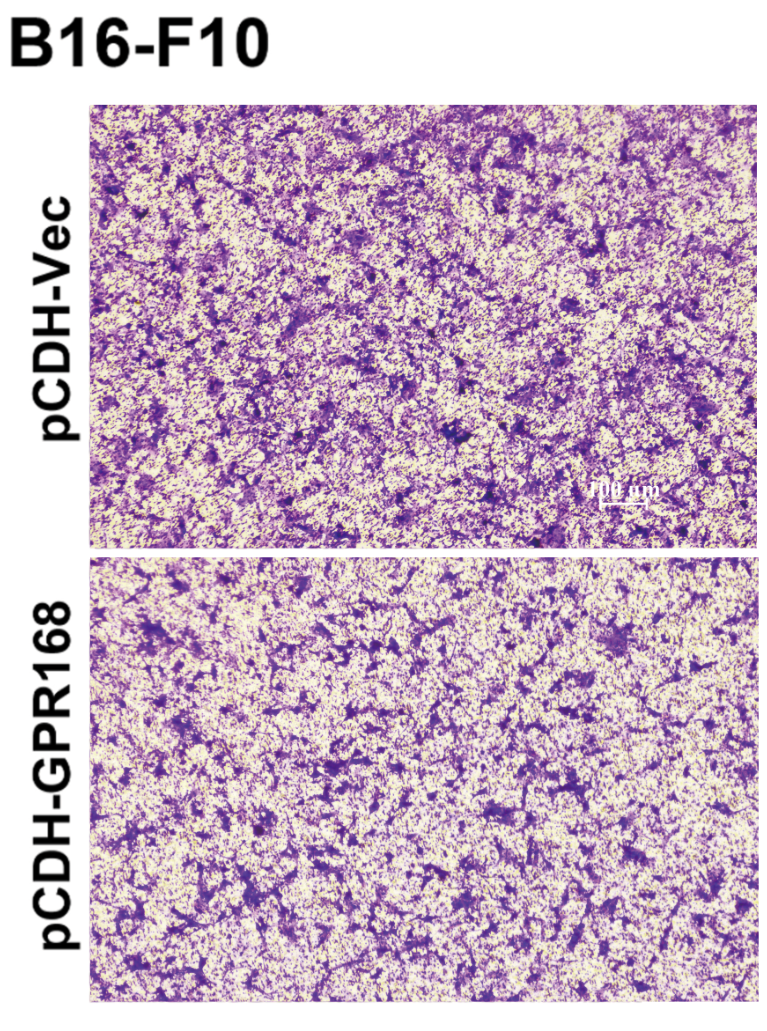

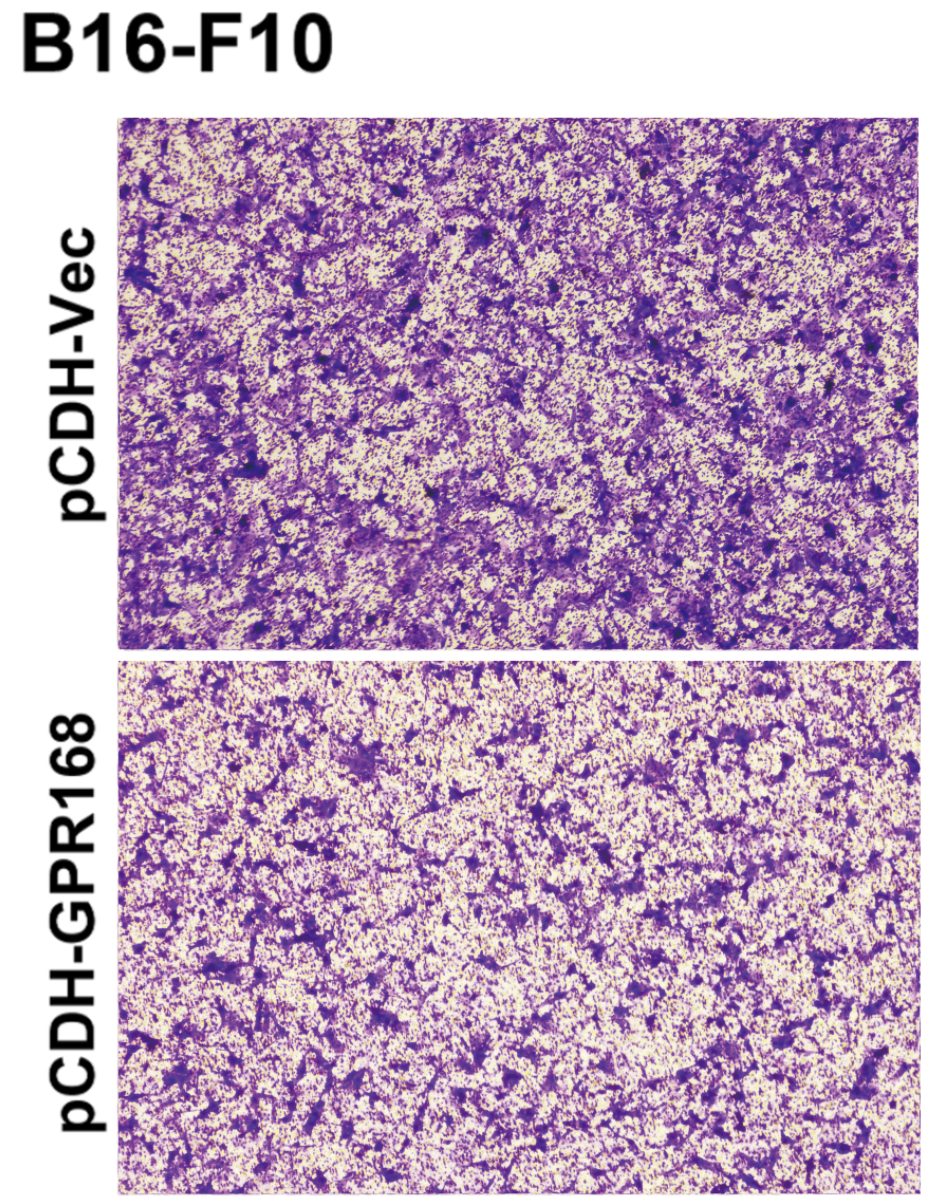


| Statistics (Fig 2H) | pCDH-Vec | pCDH-GPR168 |
| --- | --- | --- |
| 1 | 287 | 151 |
| 2 | 256 | 132 |
| 3 | 225 | 166 |
| 4 | 265 | 105 |
| 5 | 202 | 191 |

**3. Repetitions and statistics of Immunohistochemistry in Figure 3D.**


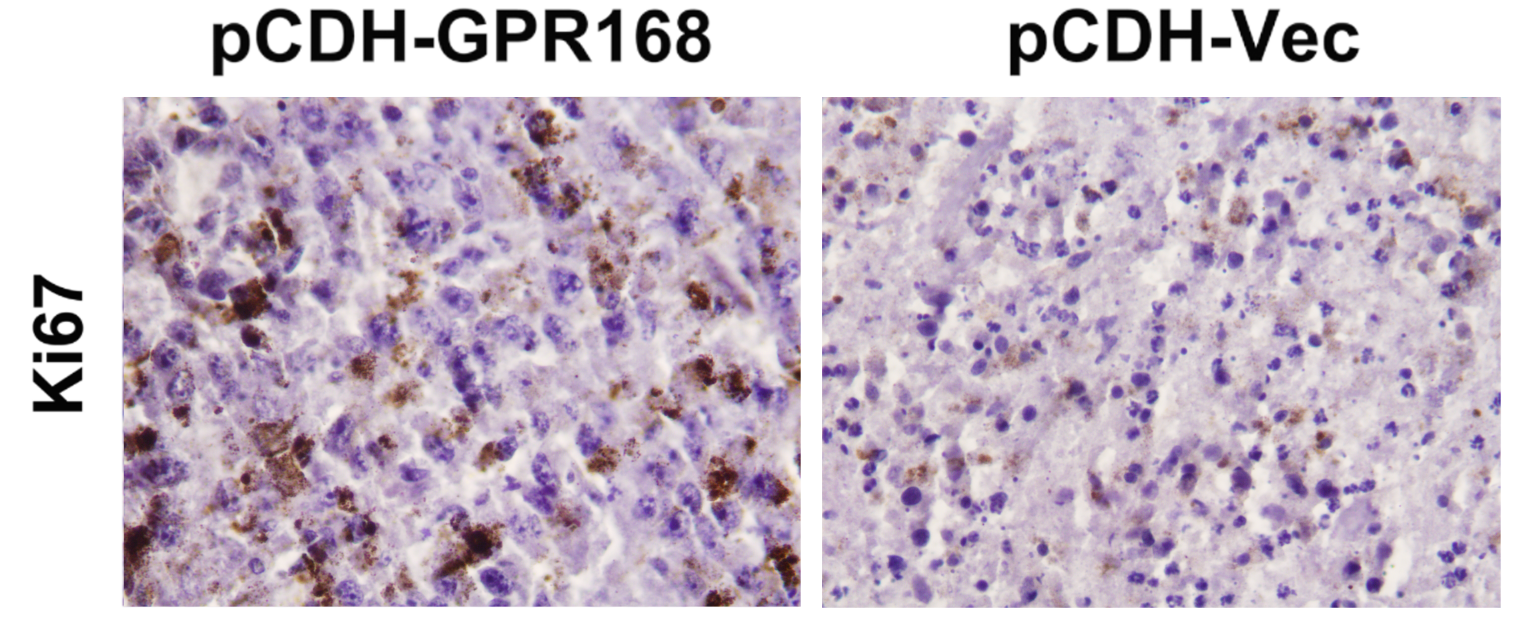

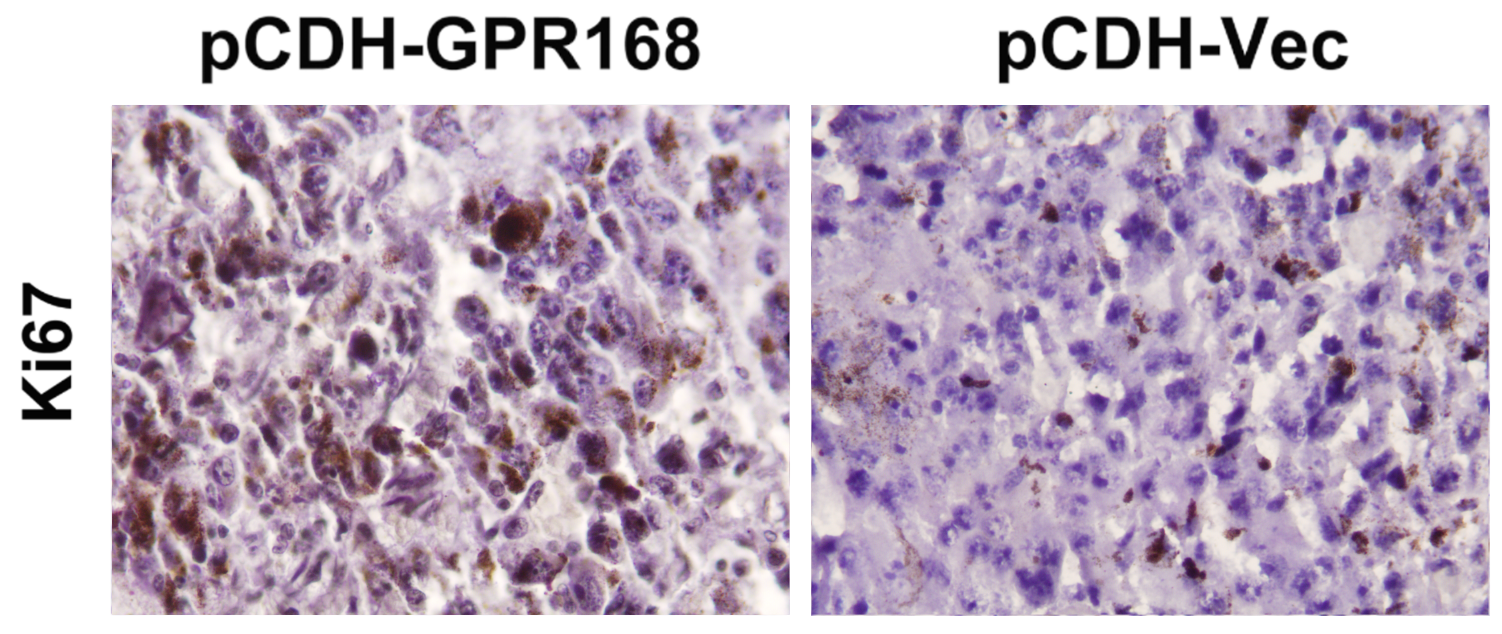

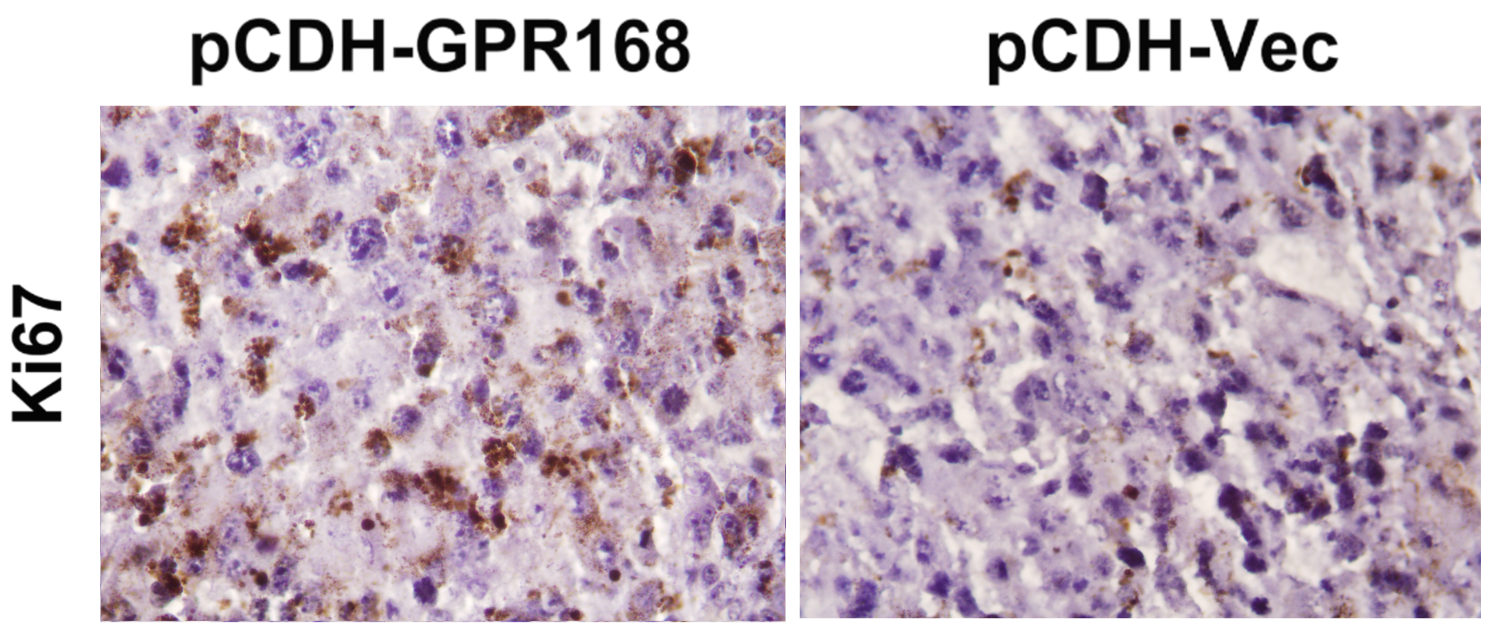

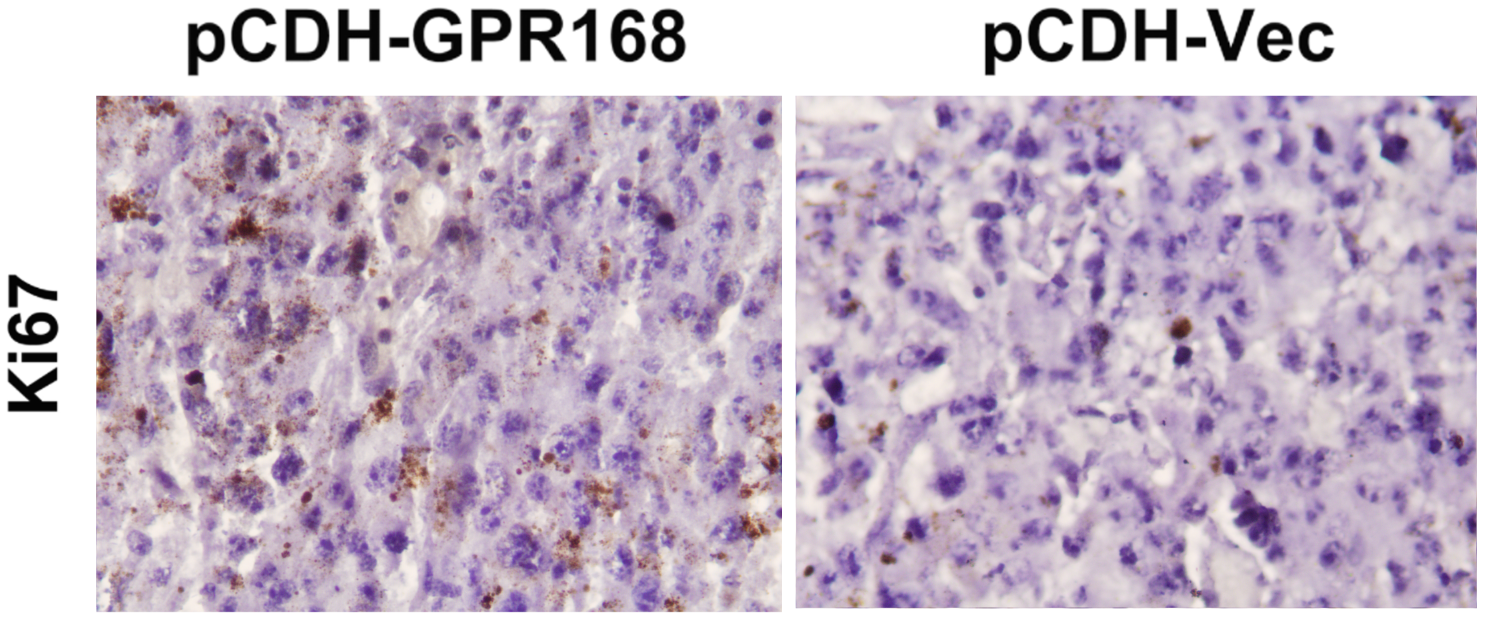


| Statistics (Fig 3D) | Cell | Hematoxylin | Ki67 | 100% |
| --- | --- | --- | --- | --- |
| 1 | pCDH-Vec | 187 | 64 | 34.22 |
|  | pCDH-GPR168 | 161 | 41 | 25.47 |
| 2 | pCDH-Vec | 195 | 64 | 32.82 |
|  | pCDH-GPR168 | 238 | 44 | c |
| 3 | pCDH-Vec | 176 | 66 | 37.50 |
|  | pCDH-GPR168 | 208 | 58 | 27.88 |
| 4 | pCDH-Vec | 184 | 55 | 29.89 |
|  | pCDH-GPR168 | 194 | 40 | 20.62 |
| 5 | pCDH-Vec | 204 | 75 | 36.76 |
|  | pCDH-GPR168 | 214 | 40 | 18.69 |

**4. Repetitions and statistics of wound healing assay in Figure 2J.**


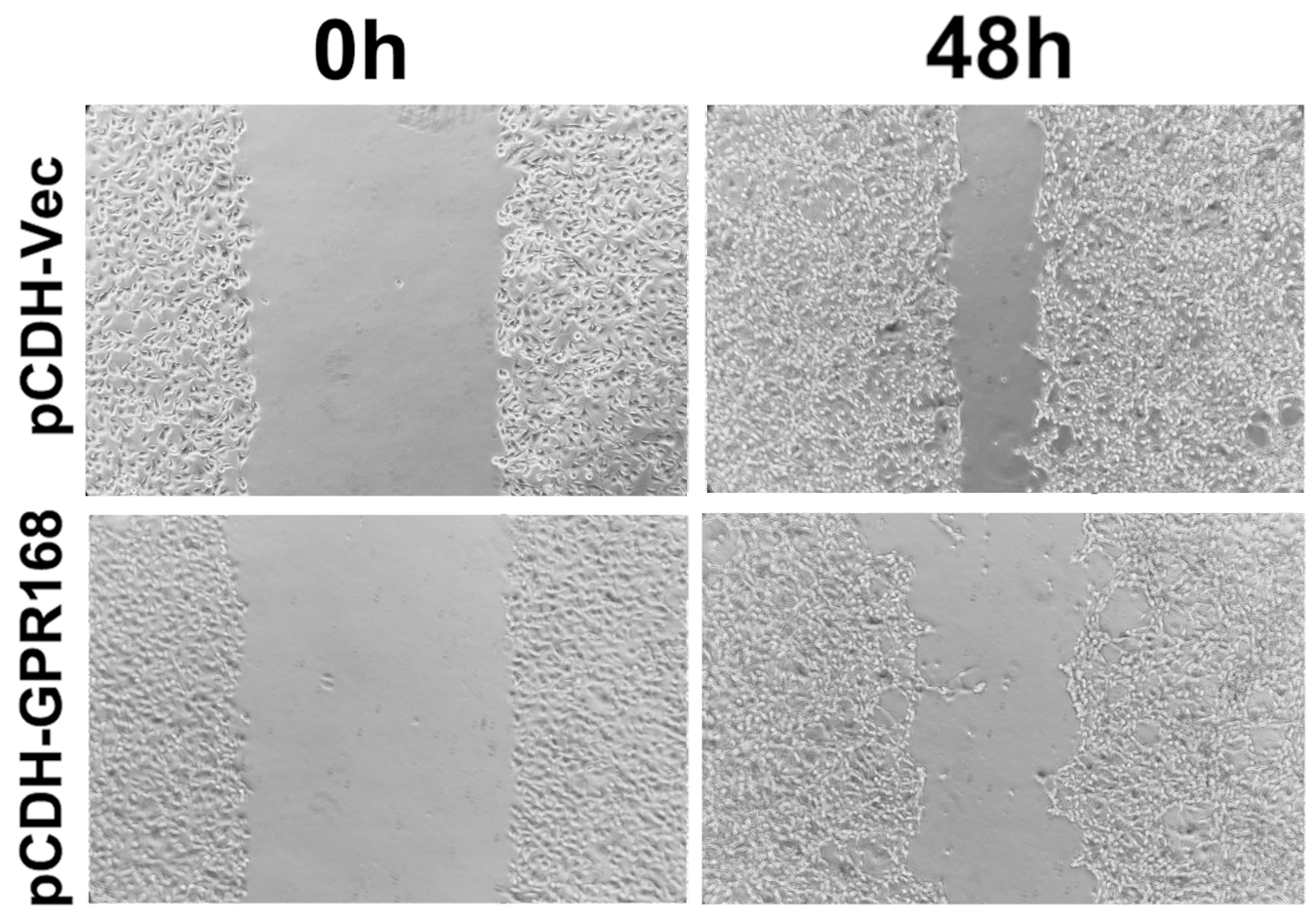

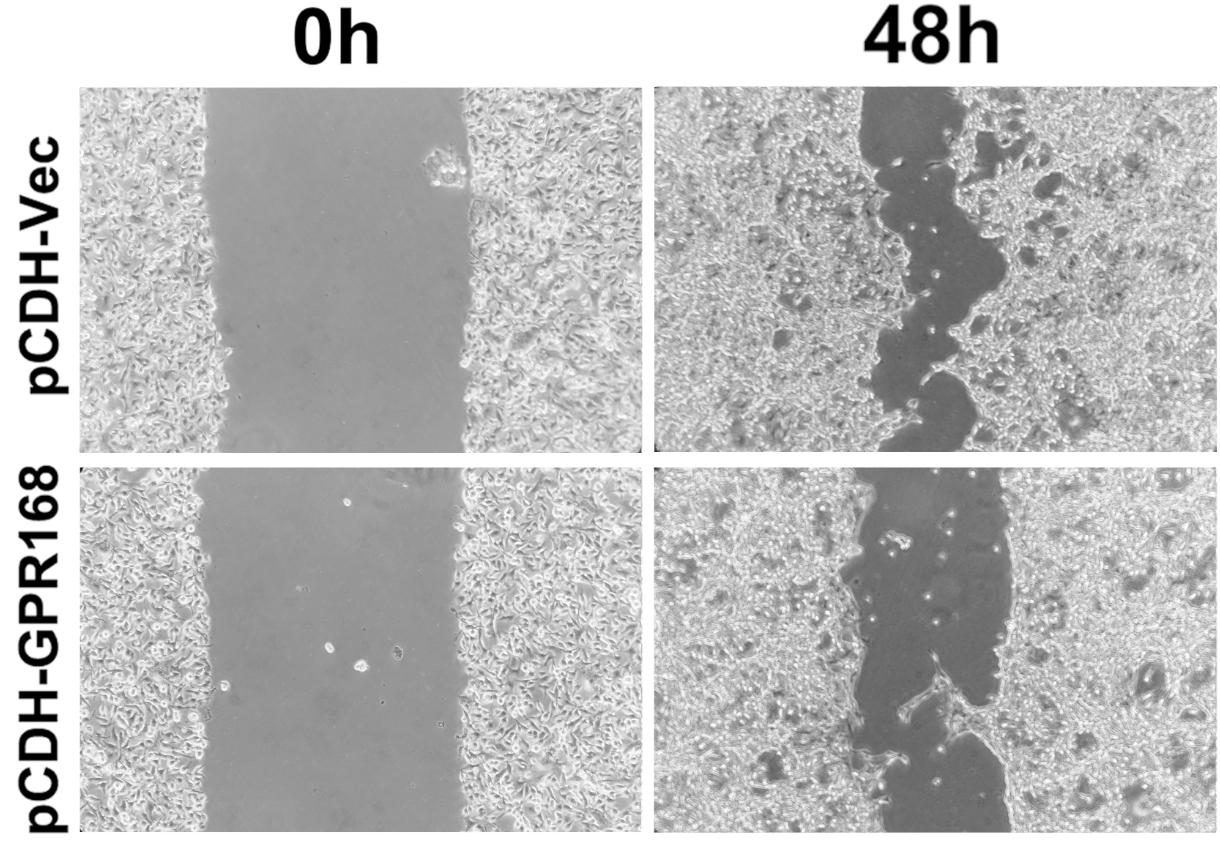

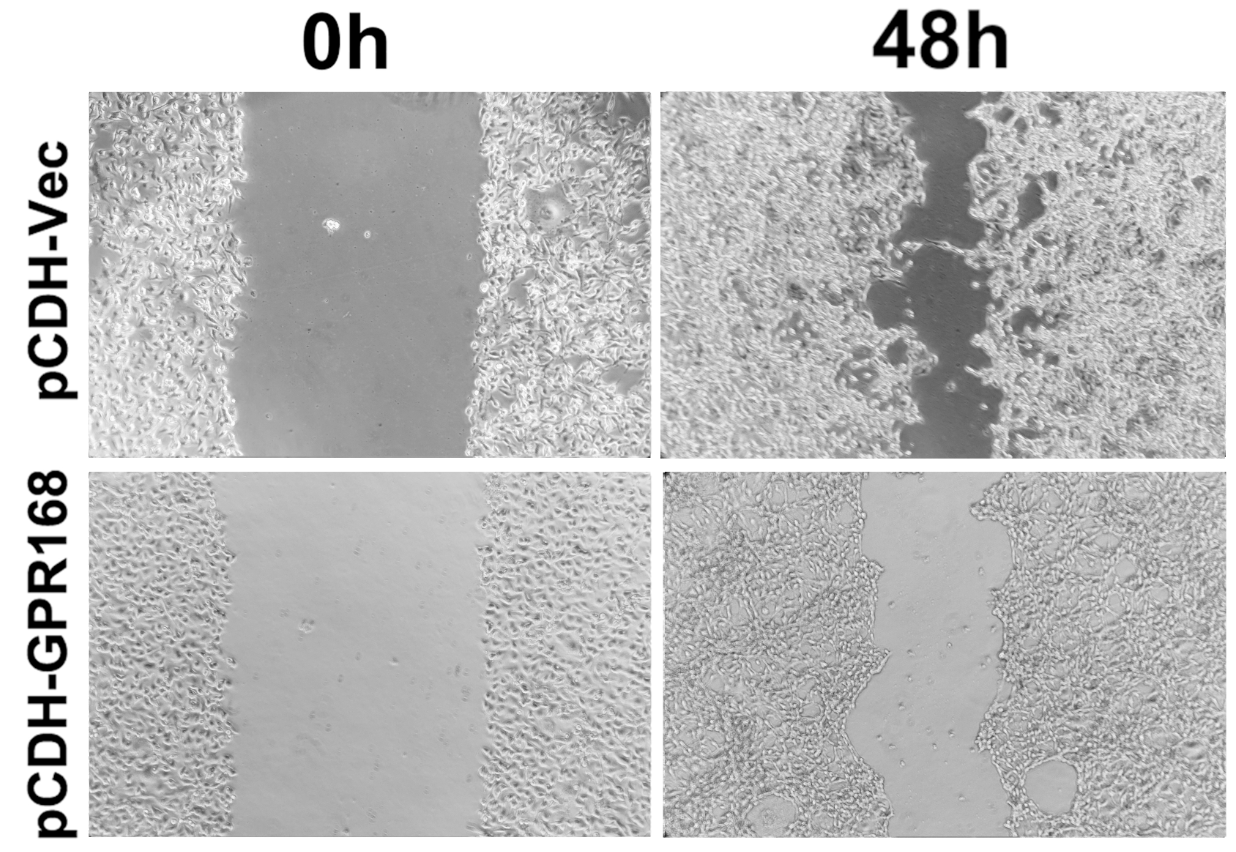

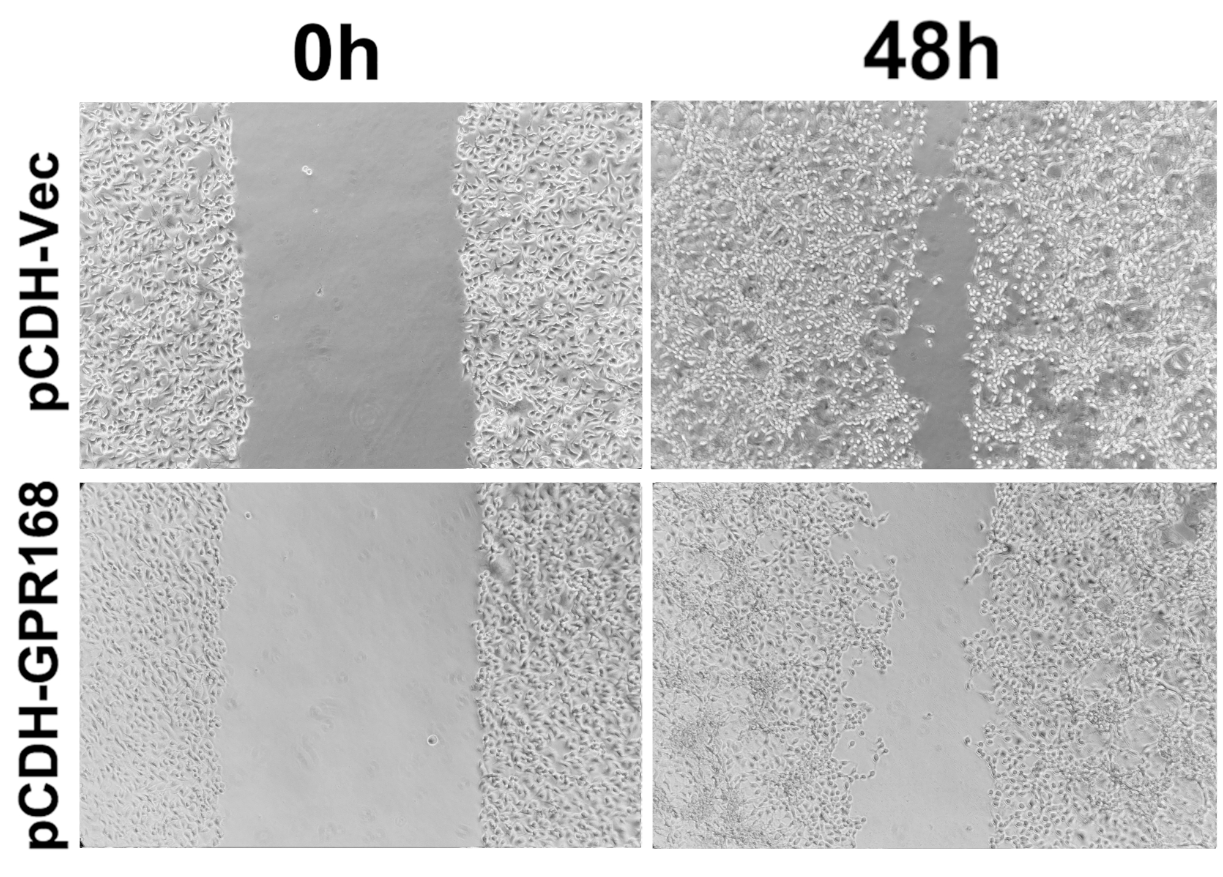


| Statistics (Fig 2J) | Cell | Would | | 100% |
| --- | --- | --- | --- | --- |
| 1 | pCDH-Vec | 20.15 | 3.73 | 18.52 |
|  | pCDH-GPR168 | 21.33 | 7.57 | 35.48 |
| 2 | pCDH-Vec | 20.96 | 4.05 | 19.34 |
|  | pCDH-GPR168 | 21.38 | 8.69 | 40.63 |
| 3 | pCDH-Vec | 19.76 | 3.71 | 18.77 |
|  | pCDH-GPR168 | 20.48 | 7.50 | 36.62 |
| 4 | pCDH-Vec | 19.84 | 4.04 | 20.35 |
|  | pCDH-GPR168 | 23.54 | 8.98 | 38.15 |
| 5 | pCDH-Vec | 20.34 | 3.25 | 15.98 |
|  | pCDH-GPR168 | 21.94 | 8.91 | 40.63 |

**5. Repetitions and statistics of Wound healing assay in Figure 4E.**


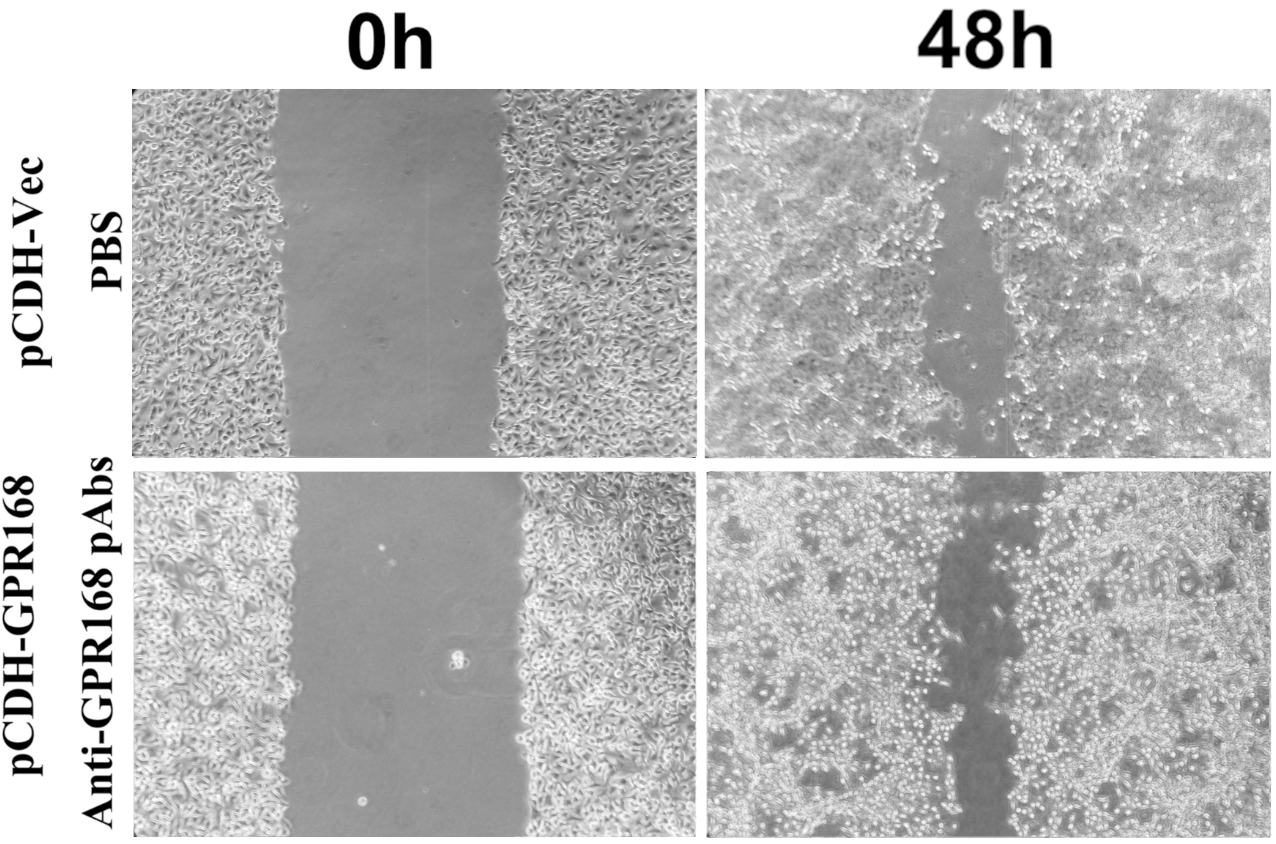

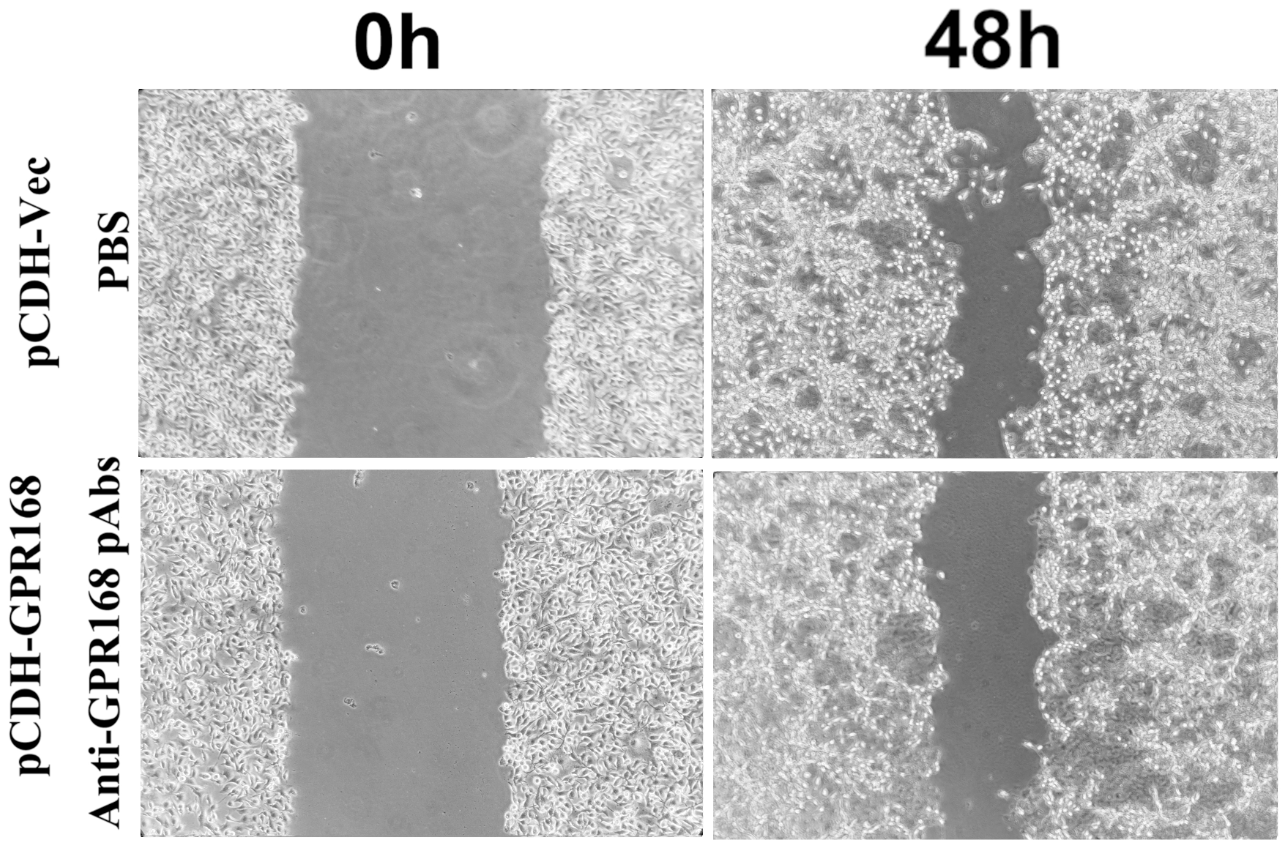

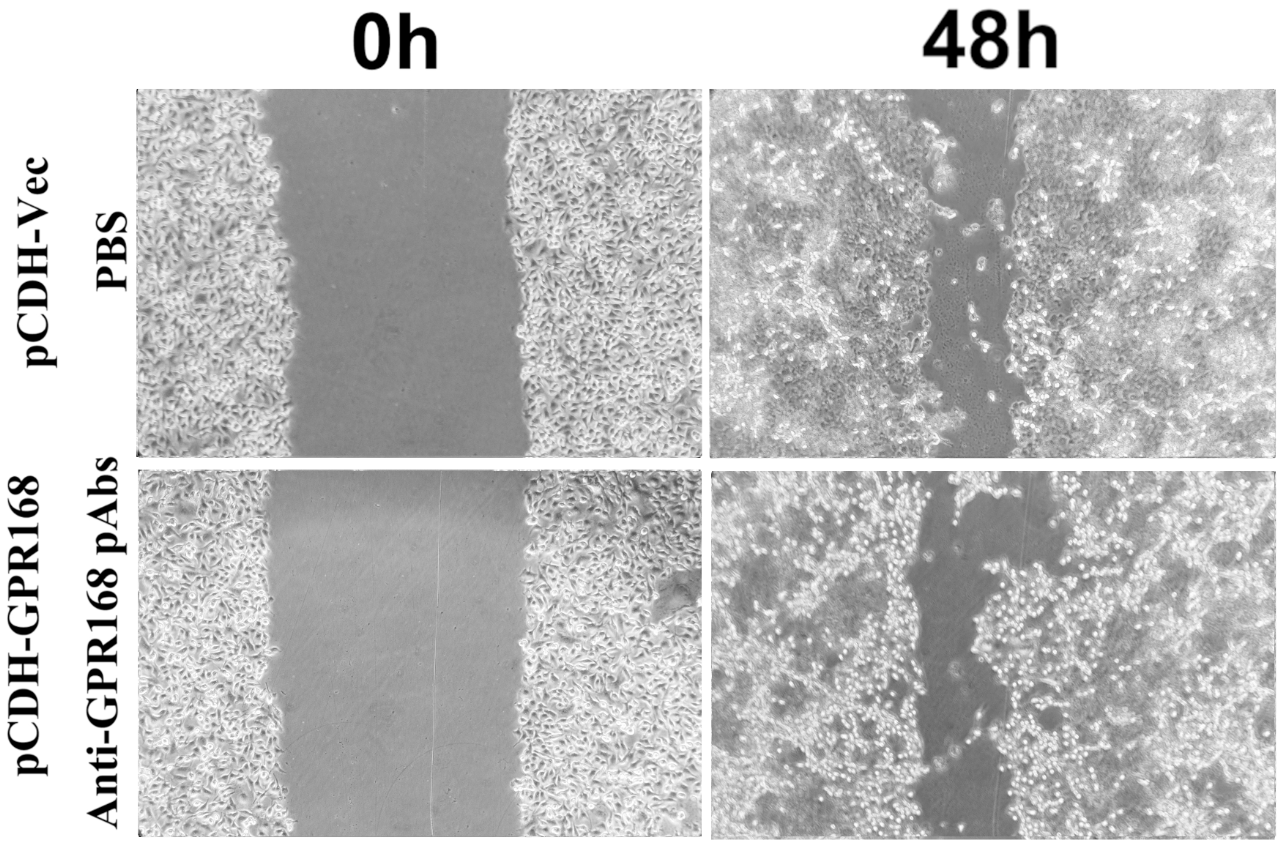

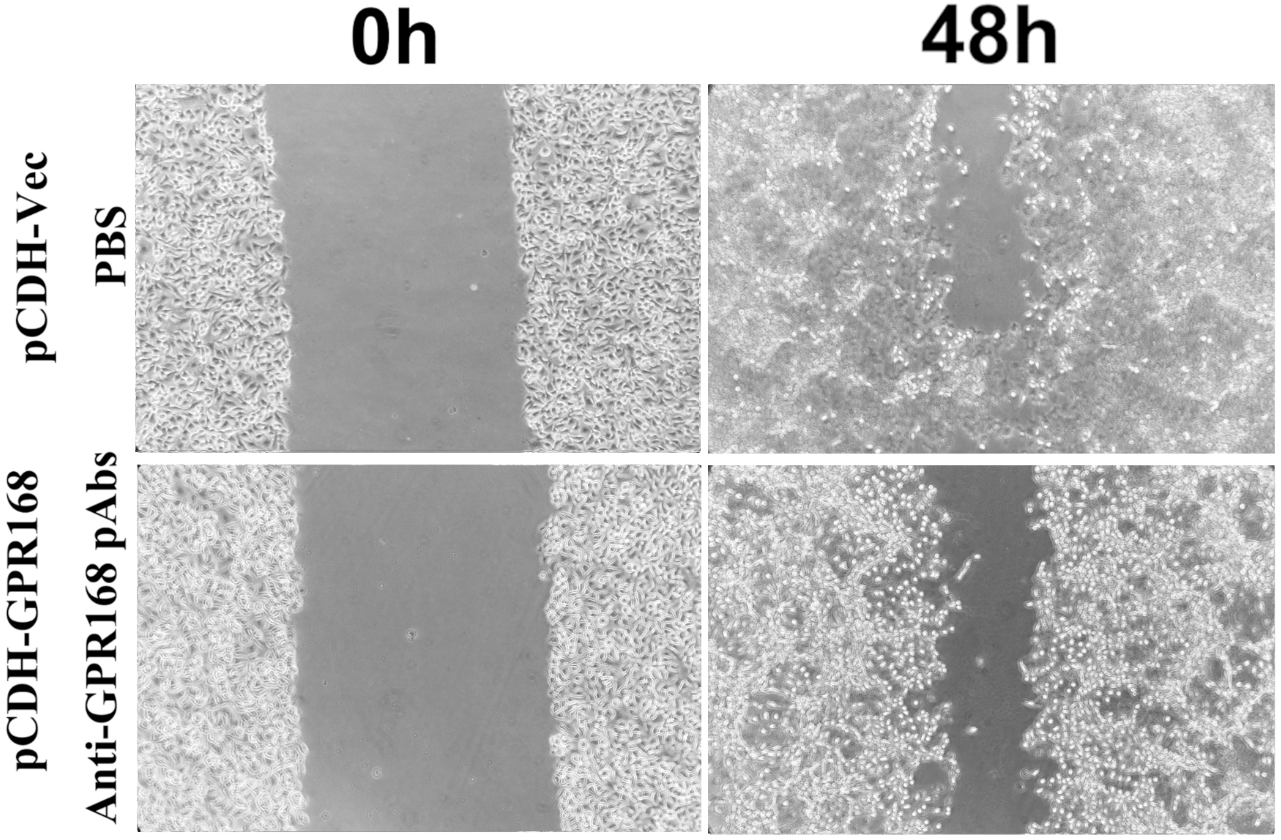


| Statistics (Fig 4E) | Cell | Would | | 100% |
| --- | --- | --- | --- | --- |
| 1 | pCDH-Vec PBS | 21.11 | 4.12 | 19.52 |
|  | pCDH-GPR168 Anti pAbs | 20.93 | 4.43 | 21.15 |
| 2 | pCDH-Vec PBS | 20.06 | 3.28 | 16.34 |
|  | pCDH-GPR168 Anti pAbs | 20.84 | 4.09 | 19.63 |
| 3 | pCDH-Vec PBS | 21.61 | 4.06 | 18.77 |
|  | pCDH-GPR168 Anti pAbs | 20.69 | 3.47 | 16.78 |
| 4 | pCDH-Vec PBS | 19.86 | 4.24 | 21.35 |
|  | pCDH-GPR168 Anti pAbs | 20.66 | 4.05 | 19.62 |
| 5 | pCDH-Vec PBS | 20.14 | 3.22 | 15.98 |
|  | pCDH-GPR168 Anti pAbs | 20.43 | 3.57 | 17.48 |
