# Supplementary material for: GPR168 functions as a tumor suppressor in mouse melanoma by restraining Akt signaling pathway
Source: PLoS One. 2024 May 28;19(5):e0302061. doi: 10.1371/journal.pone.0302061 (PMC11132440; doi:10.1371/journal.pone.0302061)
Supplement: Attachment — Submitted filename: Response to Reviewers.doc [file pone.0302061.s009.doc]

**Authors’ Responses to Editor and Reviewers’ Comments**

**Manuscript Number:** PONE-D-23-27240

**Title of Manuscript:** GPR168 functions as a tumor suppressor in mouse melanoma by restraining Akt signaling pathway

We appreciate the editor’s and reviewers’ insightful and constrictive comments on our manuscript. This document summarizes our clarifications and revisions that we have made in response to each of your comments. The line numbers highlighted in yellow in our responses indicate where we have made the changes in our revised manuscript. To help the editor and reviewers easily see our changes, we used track changes for the changes in our revised manuscript.

**Responses to Editor**

1. Please ensure that your manuscript meets PLOS ONE's style requirements, including those for file naming. The PLOS ONE style templates can be found at

https://journals.plos.org/plosone/s/file?id=wjVg/PLOSOne_formatting_sample_main_body.pdf and

https://journals.plos.org/plosone/s/file?id=ba62/PLOSOne_formatting_sample_title_authors_affiliations.pdf

**Response:** We have changed our manuscript’s style to meet PLOS ONE's style requirements.

2. We noticed you have some minor occurrence of overlapping text with the following previous publication(s), which needs to be addressed:

- https://www.nature.com/articles/s41419-018-0299-1#citeas

In your revision ensure you cite all your sources (including your own works), and quote or rephrase any duplicated text outside the methods section. Further consideration is dependent on these concerns being addressed.

**Response:** We are sorry that some of the content overlaps with the published paper. According to your review comments, we have reconsidered the duplication rate of the paper. We reviewed all the references cited in the article to make sure that each one was necessary and that each one would help us further explore the purpose and conclusions of the article. We included more original content in each paragraph to reduce the rate of duplication with existing literature. We deleted the paragraphs with repeated content in the previous article to improve the overall repetition rate of the article.

3. Thank you for stating the following financial disclosure:

"GX: the Fundamental Research Program of Shanxi Province (202203021222348), National Natural Science Foundation of China (8230089), and Scientific research start-up fund project for talent introduction of Shanxi Bethune Hospital (2023RC22). Xiang Guo designed the experiments, performed most of the experiments and wrote the paper."

Please state what role the funders took in the study. If the funders had no role, please state: ""The funders had no role in study design, data collection and analysis, decision to publish, or preparation of the manuscript.""

If this statement is not correct you must amend it as needed.

Please include this amended Role of Funder statement in your cover letter; we will change the online submission form on your behalf.

**Response:** Sorry for the unclear statement. The funders had no role in study design, data collection and analysis, decision to publish, or preparation of the manuscript. We have included this amended Role of Funder statement in cover letter. Please change the online submission form for us, thank you.

4. In your Data Availability statement, you have not specified where the minimal data set underlying the results described in your manuscript can be found. PLOS defines a study's minimal data set as the underlying data used to reach the conclusions drawn in the manuscript and any additional data required to replicate the reported study findings in their entirety. All PLOS journals require that the minimal data set be made fully available. For more information about our data policy, please see http://journals.plos.org/plosone/s/data-availability.

Upon re-submitting your revised manuscript, please upload your study’s minimal underlying data set as either Supporting Information files or to a stable, public repository and include the relevant URLs, DOIs, or accession numbers within your revised cover letter. For a list of acceptable repositories, please see http://journals.plos.org/plosone/s/data-availability#loc-recommended-repositories. Any potentially identifying patient information must be fully anonymized.

Important: If there are ethical or legal restrictions to sharing your data publicly, please explain these restrictions in detail. Please see our guidelines for more information on what we consider unacceptable restrictions to publicly sharing data: http://journals.plos.org/plosone/s/data-availability#loc-unacceptable-data-access-restrictions. Note that it is not acceptable for the authors to be the sole named individuals responsible for ensuring data access.

We will update your Data Availability statement to reflect the information you provide in your cover letter.

**Response:** All relevant data are within the manuscript and its Supporting Information files. We have revised Data Availability statement in manuscript (Lines 414-415).

5. PLOS requires an ORCID iD for the corresponding author in Editorial Manager on papers submitted after December 6th, 2016. Please ensure that you have an ORCID iD and that it is validated in Editorial Manager. To do this, go to ‘Update my Information’ (in the upper left-hand corner of the main menu), and click on the Fetch/Validate link next to the ORCID field. This will take you to the ORCID site and allow you to create a new iD or authenticate a pre-existing iD in Editorial Manager. Please see the following video for instructions on linking an ORCID iD to your Editorial Manager account: https://www.youtube.com/watch?v=_xcclfuvtxQ

**Response:** We have created a new ORCID iD: 0009-0005-6047-8238.

6. Your ethics statement should only appear in the Methods section of your manuscript. If your ethics statement is written in any section besides the Methods, please move it to the Methods section and delete it from any other section. Please ensure that your ethics statement is included in your manuscript, as the ethics statement entered into the online submission form will not be published alongside your manuscript.

**Response:** We have moved ethics statement to the Methods section (Lines 196-199).

7. PLOS ONE now requires that authors provide the original uncropped and unadjusted images underlying all blot or gel results reported in a submission’s figures or Supporting Information files. This policy and the journal’s other requirements for blot/gel reporting and figure preparation are described in detail at https://journals.plos.org/plosone/s/figures#loc-blot-and-gel-reporting-requirements and https://journals.plos.org/plosone/s/figures#loc-preparing-figures-from-image-files. When you submit your revised manuscript, please ensure that your figures adhere fully to these guidelines and provide the original underlying images for all blot or gel data reported in your submission. See the following link for instructions on providing the original image data: https://journals.plos.org/plosone/s/figures#loc-original-images-for-blots-and-gels.

In your cover letter, please note whether your blot/gel image data are in Supporting Information or posted at a public data repository, provide the repository URL if relevant, and provide specific details as to which raw blot/gel images, if any, are not available. Email us at plosone@plos.org if you have any questions.

**Response:** Our blot/gel image data are in Supporting Information.

Additional Editor Comments:

Three reviewers have carefully review your manuscript and suggested major revisions. Please read each of their comments carefully and answer each concern point by point, if necessary please also provide supporting data.

Please ensure that your decision is justified on PLOS ONE’s publication criteria and not, for example, on novelty or perceived impact.

For Lab, Study and Registered Report Protocols: These article types are not expected to include results but may include pilot data.

**Response:** Thanks for editor and reviewers’ comments. Your contributions make the manuscript much better. We have made changes and added clarifications to the manuscript based on your suggestions, which are detailed as below.

**Responses to Reviewer 1**

In this current article titled "GPR168 functions as a tumor suppressor in mouse melanoma by restraining Akt signaling pathway", authors reported the tumor suppressing properties of GPR168 in mouse melanoma. The paper contains its own merit considering the findings of a noble targets of melanoma however, it requires MAJOR REVISION before considering for publications.

**Response:** Thanks for reviewer’s comments. We have made MAJOR REVISION according to your suggestions.

1. Its unrelated to show TCGA human data set while validate with murine cell lines. Author should consider incorporation of at least one human melanoma cell lines.

**Response:** We applied Cross-Value Association Analysis (CVAA) to large-scale pan-cancer transcriptome data generated by The Cancer Genome Atlas (TCGA) [1] and found that *GPR168* is one of Differentially Expressed Genes (DEGs). Subsequently, we found that only one paper reported that GPR168 (MrgprF) acts as a tumor suppressor in cutaneous melanoma by restraining PI3K/Akt signaling pathway, and this result was confirmed by four human melanoma cells [2]. In order to further verify whether GPR168 involves in the PI3K/Akt signaling pathway, mouse melanoma B16-F10 cells were used to perform cell experiments *in vitro* and xenograph tumor models *in vivo*.

2. The data extracted from GEPIA and cBioportal even unrealistically matched with current findings as TCGA melanoma cohort published in 2015 by pancancer atlas considered both primary and metastatic tumor for their study. This cohort doesn't purely represent the story related with malignant melanoma.

**Response:** Thanks for reviewer’s professional comments. As you said, this cohort doesn't purely represent the story related with malignant melanoma. However, we want to collect as much public data as possible to make a preliminary analysis of whether GPR168 is related to melanoma. Therefore, we used as many melanoma data from multiple databases as possible to make a preliminary statistical analysis of the correlation, making it more statistical and scientific to show that GPR168 is associated with melanoma. However, this result could not prove that GPR168 is directly related to melanoma, so we performed a series of follow-up experiments *in vitro* and *in vivo* (Fig 2-4).

3. What's the rationale of overexpression total AKT expression in pCDH-GPR168 cells?

**Response:** Thanks for reviewer’s question, which allows us to discover new problems. We repeated the western blotting experiments and found that the expression of AKT in pCDH-Vec and pCDH-GPR168 had no significant difference (Fig 3F).

**Fig 3.** Overexpression of GPR168 inhibited xenograft tumors growth *in vivo*. (A) The sizes of allogenic tumors at day 30 were smaller by pCDH-GPR168, compared to those by pCDH-Vec. (B) GPR168 overexpression significantly reduced MM tumor growth in nude mice by tumor volume examination from 0 to 30 day. (C) Overexpression of GPR168 significantly suppressed the weights of allogeneic tumors. (D, E) the positive cells of Ki67 in allogenic tumor tissues. Scale bar is 100 µm, (E) Quantification for (D). (F) Akt, p-Akt, GSK-3β, p- GSK-3β, β-catenin, Myc, CyclinD1, CDK4 protein expression in pCDH-GPR168 group and pCDH-Vec group (Western blotting). (G) *β-catenin*, *Myc*, *CyclinD1*, *CDK4* mRNA expression in pCDH-GPR168 group and pCDH-Vec group cells (RT-qPCR). (H) A signaling pathway model for GPR168 function in B16-F10 cell line. β-actin as normalized protein. **P* < 0.05, ***P* < 0.01, ****P* < 0.001, *t-*test.

4. After blocking the function of GPR168, p-AKT expression didn't change and cell viability didn't alter compared with empty vector. How this target can be trusted to became a control factor of tumorigenesis?

**Response:** The overexpression of GPR168 could inhibit the proliferation and migration of melanoma, as well as the formation of xenograft tumors (Fig 2). After a series of functional experiments, it was found that GPR168 could function through the AKT signaling pathway (Fig 3). We preliminarily concluded that *GPR168* is a tumor suppressor (melanoma) gene. Subsequently, we performed rescue experiment, and found that Anti-GPR168 pAbs could block the function of pCDH-GPR168 plasmid in B16-F10 tumor cells (Fig 4), which further confirmed that GPR168 could inhibit the tumorigenesis of melanoma through AKT signaling pathway. Taken together, we conclude that GPR168 could be a control factor of tumorigenesis (melanoma).

5. The figures look blurred.

**Response:** Thanks for reviewer’s comments. We repeated Western blotting experiment and re-made the Fig 1D, 2B, 3F and 4C.

**Responses to Reviewer 2**

Although the bioinformatics analysis shows a decreased expression of GPR168 across different human tumors including melanoma, the paper falls short of showing a causality of low expressed GPR168 on melanoma. The entire paper relied on one mouse melanoma cell line (B16-F10) with or without ectopic expression of GPR168. There are inconsistencies in the results even with this cell line. The authors mistakenly considered injecting mouse melanoma cell line F10 into nude mice an allogeneic model.

**Response:** Thanks for reviewer’s comments. In this paper, we found that the expression of GPR168 might be correlated with the tumorigenesis of melanoma (Fig 1). Subsequently, we found that one paper reported that GPR168 (MrgprF) acts as a tumor suppressor in cutaneous melanoma by restraining PI3K/Akt signaling pathway, and this result was confirmed by four human melanoma cells [2]. In order to further explore the function of GPR168 in melanoma cells, cell experiments *in vitro* (Fig 2) and xenograph tumor models *in vivo* (Fig 3) were performed and the results showed the overexpression of GPR168 could inhibit the tumorigenesis of melanoma and GPR168 involved in the PI3K/Akt signaling pathway. Furthermore, we confirmed this conclusion by rescue experiment (Fig 4).

**Responses to Reviewer 3**

The manuscript “GPR168 functions as a tumor suppressor in mouse melanoma by restraining Akt signaling pathway” by Guo et al is very interesting as there are only a few tumor suppressors. Please see below my specific comments.

Specific Comments:

1. Expression of GPR in B10-F10 should be shown by immunoblotting. Otherwise, we assume that it is there without any proof.

**Response:** Thanks for reviewer’s comments. We have performed Western blotting as reviewer’s suggestion. The western blotting results showed that the B16-F10 cell line with stable GPR168 expression was successfully constructed (Fig 2B).

**Fig 2. Overexpression of GPR168 inhibited B16-F10 cell proliferation and migration.** (A, B) The efficiency of pCDH-GPR168 (GPR168 overexpression) was verified by RT-qPCR and western botting, compared to scramble pCDH-Vec. (C, D) GPR168 overexpression inhibited cell proliferation in B16-F10 cells by Ki67 Immunofluorescence assay. Scale bar is 100 µm. (E, F) Result of CCK-8 assay (E) and Growth Curve (F) showed that overexpression of GPR168 significantly inhibited B16-F10 cell proliferation. (G, H) overexpression of GPR168 decreased the migration ability of B16-F10 by transwell assay at OD570. Scale bar is 100 µm. (I, J) Wound healing assay showed that overexpression of GPR168 inhibited the migration of B16-F10 cells. Scale bar is 100 µm. **P* < 0.05, ***P* < 0.01, ****P* < 0.001, *t*-test.

2. CDK4 is down but no change in cyclin D1. Also, how then there is a decrease in Ki67?

**Response:** Thanks for reviewer’s question, which allows us to discover new problems. We repeated the western blotting experiments and found that both of CDK4 and cyclin D1 protein were decreased (Fig 3F).

**Fig 3. Overexpression of GPR168 inhibited** **xenograft tumors growth *in vivo*.** (A) The sizes of allogenic tumors at day 30 were smaller by pCDH-GPR168, compared to those by pCDH-Vec. (B) GPR168 overexpression significantly reduced MM tumor growth in nude mice by tumor volume examination from 0 to 30 day. (C) Overexpression of GPR168 significantly suppressed the weights of allogeneic tumors. (D, E) the positive cells of Ki67 in allogenic tumor tissues. Scale bar is 100 µm, (E) Quantification for (D). (F) Akt, p-Akt, GSK-3β, p- GSK-3β, β-catenin, Myc, CyclinD1, CDK4 protein expression in pCDH-GPR168 group and pCDH-Vec group (Western blotting). (G) *β-catenin*, *Myc*, *CyclinD1*, *CDK4* mRNA expression in pCDH-GPR168 group and pCDH-Vec group cells (RT-PCR). (H) A signaling pathway model for GPR168 function in B16-F10 cell line. β-actin as normalized protein. **P* < 0.05, ***P* < 0.01, ****P* < 0.001, *t-*test.

3. Minor Comments: Figures are of poor quality especially IHC. Effort must be made to produce high quality figures.

**Response:** Sorry for poor quality of figures. We have made revise in Fig 3D.

**References:**

[1] Li QG, He YH, Wu H, Yang CP, Pu SY, Fan SQ, et al. A normalization-free and nonparametric method sharpens largescale transcriptome analysis and reveals common gene alteration patterns in cancers. Theranostics. 2017;7(11):2888-2899. https://doi.org/10.7150/thno.19425. eCollection 2017 PMID: 28824723

[2] Shen QS, Han YF, Wu K, He YM, Jiang XL, Liu PS, et al. MrgprF acts as a tumor suppressor in cutaneous melanoma by restraining PI3K/Akt signaling. Signal Transduct Target Ther. 2022;7(1):147. Epub 2022/05/04. https://doi.org/10.1038/s41392-022-00945-9 PMID: 35504869
