# Supplementary material for: GPR168 functions as a tumor suppressor in mouse melanoma by restraining Akt signaling pathway
Source: PLoS One. 2024 May 28;19(5):e0302061. doi: 10.1371/journal.pone.0302061 (PMC11132440; doi:10.1371/journal.pone.0302061)
Supplement: Attachment — Submitted filename: Response to Reviewers.doc [file pone.0302061.s010.doc]

**Authors’ Responses to Editor and Reviewers’ Comments**

**Manuscript Number:** PONE-D-23-27240

**Title of Manuscript:** GPR168 functions as a tumor suppressor in mouse melanoma by restraining Akt signaling pathway

We appreciate the editor’s and reviewers’ insightful and constrictive comments on our manuscript. This document summarizes our clarifications and revisions that we have made in response to each of your comments. The line numbers highlighted in yellow in our responses indicate where we have made the changes in our revised manuscript. To help the editor and reviewers easily see our changes, we used track changes for the changes in our revised manuscript.

**Responses to Reviewers**

1. Is the manuscript presented in an intelligible fashion and written in standard English?

PLOS ONE does not copyedit accepted manuscripts, so the language in submitted articles must be clear, correct, and unambiguous. Any typographical or grammatical errors should be corrected at revision, so please note any specific errors here.

Reviewer #4: No

**Response:** We feel sorry for our poor writings, however, we do invite a friend of us who is a native English speaker from the USA to help polish our article. And we hope the revised manuscript could be acceptable for you.

2. Data point distribution for all graphs should be included in each figure.

**Response:** Thanks for the comment. We have now included data point distribution in all graphs.

3. Showing representative western immunoblots is not enough, data needs to be normalized, graphed, and individual data points must be shown.

**Response:** Thanks for your suggestion. We have now normalized western blot data and showed in Fig 1E, Fig 2B, Fig 3H, Fig 4D.

4. Limitations of this study must be stated in the discussion i.e human bioinformatics data used but only mouse data from one cell-line included and no human data included. Additionally, the limitations identified by the other reviewers must be stated.

**Response:** Thanks for the comment. We have now pointed this limitation in discussion (Lines 359-360).

5. The comment made by the authors, “We repeated the western blotting experiments and found that both of CDK4 and cyclin D1 protein were decreased (Fig 3F).” based on reviewer 3’s comments of the fact that cyclin D1 did not decrease this is quite concerning since it suggest reproducibility issues which is why the authors should not show just the representative western immunoblots but rather both representative western immunoblots and quantifications of the western immunoblots normalized.

**Response:** Thanks for your comments. We repeated three times each western blot. We have now showed all western blot results in Supporting information and quantifications of the western immunoblots normalized in Fig 1E, Fig 2B, Fig 3H, Fig 4D.
